# Evaluation of molecular effects associated with apoptosis, tumour progression, angiogenesis and metastasis by a novel combination of drugs with ormeloxifene in triple negative breast cancer cells

**DOI:** 10.37349/etat.2024.00235

**Published:** 2024-06-11

**Authors:** Shehna Sharaf, Sreelekshmi S, Saikant Regidi, Abi Santhosh Aprem, Rajmohan Gopimohan, Lakshmi S

**Affiliations:** Institute of Experimental Endocrinology and Oncology "G. Salvatore"-National Research Council (IEOS-CNR), Italy; Corporate Research and Development Centre, HLL Lifecare Limited, Thiruvananthapuram 695 017, Kerala, India

**Keywords:** Ormeloxifene, metastasis, breast cancer, tumour

## Abstract

**Aim:**

To investigate the molecular effects of a novel combination [sertraline and plumbagin (comb) with ormeloxifene (Orm)] for anticancer activity in triple negative breast cancer cell line “MDA-MB-231”.

**Methods:**

The cytotoxic effect of the drugs was analyzed by the MTT assay and nuclear morphological changes by acridine orange/ethidium bromide (AO/EB) staining. Induction of apoptosis by annexin V-FITC staining, active caspase-3 detection and cell cycle analysis were studied in vitro on “MDA-MB-231” cells. The qRT-PCR was done to explore the upregulation and down regulation of targeted genes for angiogenesis, metastasis, tumor suppression and protein folding on the triple negative breast cancer cells. The preliminary anti-angiogenic effect of the drugs was assessed by chorioallantoic membrane (CAM) assay.

**Results:**

Orm showed inhibitory effects in “MDA-MB-231” cells in a dose and time dependent manner whereas; the drugs in combination gave better cytotoxic effects in the screening MTT assay. Orm + comb was more effective than Orm alone in eliciting apoptosis as well as inhibited the single cell to grow into a colony. CAM assay using Orm and Orm + comb suggested the anti-angiogenic potential which was further confirmed by the downregulation of *VEGF* in “MDA-MB-231” cells by qRT-PCR studies. The combination was found to effectively upregulate the expression of *P53* and *P21* and downregulate the gene expression of zinc finger E-box binding homeobox 1 (*ZEB1*) and heat shock protein 70 (*HSP70*) in “MDA-MB-231” cancer cells.

**Conclusions:**

Collectively this study reveals the efficacy of Orm + comb as more significant than the clinically used tamoxifen (Tam). The study elucidates the promising novelty of the combination as a potential chemotherapeutic intervention for mitigating the aggressiveness of triple negative breast cancer and it addresses the intrinsic resistance caused by single drug treatments.

## Introduction

Breast cancer is a frequent malignancy with a dormant and insidious character which grows slowly, but with advanced metastasizing properties. Despite current therapies significantly delaying tumor progression, there is a chance of recurrence dependent on several factors such as tumor stage, metastasis, and multidrug resistance, which may contribute to high mortality rates [1]. The development of breast cancer is a multi-step process involving multiple cell types and its prevention remains always challenging. The majority of deaths from breast cancer are due to the result of metastasis to other organs in the body and hence metastatic breast cancer is still considered an incurable disease. Chemotherapy remains the main treatment modality for cancer patients, but the morbidity and mortality rates in patients with metastatic cancer remain high [2]. The limitations in the existing methods of treatment including toxicity to other tissues warrant the need for alternatives that could remove whole cancer without damaging other tissue of the body.

Centchroman (CC) also known as ormeloxifene (Orm) belongs to the selective estrogen receptor modulator (SERM) class of compounds that selectively induces apoptosis in human breast cancer cells and helps in downregulating *VEGF* induced angiogenesis by modulating vascular *VEGF* receptor 2 (*VEGFR2*) pathway and can be used as a potential therapeutic drug [3]. Anti-proliferative effect of Orm is reported to inhibit colony forming efficiency of head and neck squamous cell carcinoma (HNSCC) cells by modulating PI3K/mTOR pathway [4]. Orm is also reported to arrest cell cycle in G0–G1 phase via modulation of regulatory proteins [such as induction of *P21/P27* and inhibition of myeloid cell leukemia-1 (Mcl-1), cyclin D1 and cyclin-dependent kinase 4 (CDK4)], which helps to induce apoptosis, inhibit growth and metastatic potential of prostate cancer cells [5]. Further Orm is also reported to inhibit cell growth and induce apoptosis in ovarian cancer cells, including ovarian cancer cell lines resistant to cisplatin [6].

In recent years, plant-based medicines have drawn much attention for cancer treatments due to their promising efficacy and low toxicity compared with conventional chemotherapeutic drugs. Plumbagin selected for the synergistic effect with the Orm is the main phytochemical present in *Plumbago indica* and is reported to exert anticancer effects via multiple mechanisms. The anticancer activity of plumbagin was found to be associated with alternations in apoptosis [Bcl-2 and Bcl-2-associated X protein (Bax)] and autophagy [microtubule-associated protein 1A/1B-light chain 3 (LC3-1), LC3-phosphatidylethanolamine conjugate (LC3-II) and Beclin I]. The phytotherapeutic treatment using plumbagin is reported to target lung cancer through the ROS and intrinsic mitochondrial apoptotic pathway [7]. The anticancer effect of plumbagin is also reported due to the presence of pleiotropic nature, by targeting several molecular mechanisms including apoptosis, cell cycle arrest, pathways of autophagy, anti-angiogenesis, anti-invasion and anti-metastasis [8]. Plumbagin is described to be a potent inducer of ROS, suppressor of cellular glutathione and has the ability to re-sensitize the chemo and radio-resistant cancer cells when used in combination or alone [9]. Plumbagin can inhibit expansion and instigate apoptosis of hepatic cancer through inhibition of the expression of apoptosis-related proteins and subsequently down regulate the mTOR signalling pathway [10]. Plumbagin inhibited tube formation of human endothelial progenitor cells and *VEGF* induced migration without cytotoxic effects. Plumbagin inhibited angiogenesis via phospholipase C (PLC), protein kinase B (PKB), extracellular signal-regulated kinase (ERK) signalling pathways [11].

Sertraline hydrochloride is another drug which has added in the combination, which can be encapsulated by liposomes and other vectors or it can be used as a free drug [12]. Sertraline, belong to the selective serotonin reuptake inhibitor (SSRI) class of antidepressants and has shown to have anti-proliferative effects on various human cancer cell lines, such as colon, prostate and breast cancer [13]. Treatment of patients with sertraline at high doses may be at risk of hepatotoxicity due to induced morphometric changes, provoked histological and histochemical alterations like hepatocytes hydropic degeneration, necrosis, nuclear alteration, sinusoidal dilation, bile duct hyperplasia, portal fibrosis, etc. [14]. The studies highlighted a significant inhibition of angiogenesis and tumorigenesis through the use of sertraline [15]. Sertraline demonstrated its potential to sensitize drug-resistant gastric cancer cells. A comprehensive investigation involving thirty derivatives of sertraline was conducted to evaluate their anti-cancer effects and the most promising compound exhibited a compelling inhibitory concentration (IC_50_) value of 5.2 µmol/L. It was also reported to induce apoptosis induction and cell cycle arrest after the treatment of drug-resistant gastric cancer cells with sertraline [16].

Tamoxifen (Tam) is the currently used clinical drug belonging to the SERM class of compounds to treat estrogen responsive breast cancers. Orm is another novel SERM which is reported to be more non-toxic and tolerable with fewer side effects. It’s been used for treatment of abnormal uterine bleeding and for contraception for almost two decades. In this study, an attempt was made to reposition Orm as a potential alternative to Tam for the treatment of breast cancer. Recognizing the inherent drug resistance of single drug especially in breast cancer, a novel combination was tried using Orm so that the drugs act through multiple pathways and bypass the resistance mechanisms. In the present study, Tam was compared with Orm and the Orm + comb (sertraline and plumbagin). Adriamycin (Adr) otherwise called doxorubicin was used as positive control. The findings were compared with the efficacy of Adr and Tam for assessing the novel therapy, evaluating the combinatorial approach, diversity mechanisms, clinical relevance, safety and tolerability.

The combined action or synergism is expected to give more potent anti-cancer effects to Orm rather than when used alone. The synergism will overcome inherent drug resistance which use to follow after some course of treatment and is expected to have broad spectrum coverage. The synergistic effects of the combination vs. Orm were evaluated for clonogenic and anti-angiogenisis activity. The down regulation and upregulation studies of potential genes for metastasis, angiogenesis, protein folding and tumor suppression were studied using real-time PCR. Apoptosis inducing effects and cell cycle studies were studied using flow cytometry. The results were compared with the efficacy of Adr and Tam.

## Materials and methods

### Culture of cell lines and treatment

“MDA-MB-231” cells were procured from National Centre for Cell Sciences (NCCS) Pune, authenticated by DNA profiling using short tandem repeats (STRs) (16 STR loci).

“MDA-MB-231” cells were passaged over two times in a week and confirmed mycoplasma free on regular basis by Hoechst staining. Mycoplasma free cells in the exponentially phase of the 8th–10th passage were taken for all the analysis. Dermal fibroblast cells (juvenile foreskin) from Hi-media laboratories, India which were used for the studies. The cells were cultured in Dulbecco’s modified Eagle’s medium (DMEM) with 10% heat-inactivated fetal bovine serum (FBS) and antibiotics. The cells were grown at 37℃ in a humidified 5% CO_2_ atmosphere. Media and supplements were purchased from Gibco (Life Technologies, Thermo Fisher Scientific). All treatments were done with Orm, Adr, Tam and the combination (Orm + comb) in final concentrations of 12.5 µmol/L, 25 µmol/L and 50 µmol/L.

### Cell viability assay using MTT

Cell viability of cells grown in monolayer was assessed using the colorimetric, formazan-based MTT assay (Merck). Cells were seeded in 96-well plates (5,000 cells/well) and adherent cells were left to attach for 24 h prior to treatment with Orm final concentration of 3.125–50 µmol/L in double dilutions for 24 h, 48 h, 72 h and 144 h. The solvent used was DMSO and the ratio of each drug in the combination of Orm:sertraline:plumbagin was 1:1:1. The concentrations were so adjusted that the cumulative concentration of the combinations were from 3.125–50 µmol/L. After the specified incubations, the absorbance was measured at 570 nm using ELISA reader (Tecan infinite M200 PRO) and the percentage of cytotoxicity was calculated and the results are presented, with blank subtracted, as percent relative to untreated control ± standard deviation (SD) of the mean [17].


$$\text{Cytotoxicity }(\text{\%})\;=\;100\;–\;[(\frac{\text{Optical density }(\text{OD})\text{ of treated cell}}{\text{OD of negative control}})\times 100]$$

### Nuclear morphological analysis using acridine orange/ethidium bromide staining

Briefly, “MDA-MB-231” cells were seeded in 96 well plates at seeding density of 5 × 10^5^ cells and treated with different concentrations of Orm (25 µmol/L and 50 µmol/L) and the combination/formulation (25 µmol/L and 50 µmol/L) for 48 h. After washing once with PBS the cells were stained with 100 μL of a mixture (1:1) of acridine orange (AO; sigma A9231) ethidium bromide (EB; sigma e7637) at (4 μg/mL) solutions in 96 well plate. The cells were immediately washed with PBS and observed under a fluorescence microscope at 450–490 nm [18].

### Evaluation of induction of apoptosis

#### Caspase-3 expression

Cells were treated with Orm and Orm + comb (25 µmol/L and 50 µmol/L), Tam (25 µmol/L and 50 µmol/L) and Adr for 72 h and 144 h. The expression of caspase-3 was analyzed using FITC conjugated monoclonal active caspase-3 antibody apoptosis detection kit [Becton, Dickinson and Company (BD) Pharmingen, cat no: 550480] and the results were interpreted using Diva software analysis [19].

#### Annexin V Status

To evaluate the annexin V-positivity, cells were treated with different concentrations of Orm, Orm + comb (25 µmol/L and 50 µmol/L), Tam (25 µmol/L and 50 µmol/L) and Adr for 48 h. Early time point of 48 h was selected for the study because annexin V expression is an early event in apoptosis. The percentage of cells actively undergoing apoptosis were analyzed using annexin V-FITC apoptosis detection kit II (BD Pharmingen, cat no: 556547) by flow cytometry and the results were analysed using cell quest pro software analysis [20].

#### Cell cycle analysis

The phase of the cell cycle at which treated cancer cells get arrested was determined using flow cytometry [Fluorescence-activated cell sorting (FACS) Becton Dickinson]. The cells were treated with different concentrations of Orm, Orm + comb (25 µmol/L and 50 µmol/L), Tam (25 µmol/L and 50 µmol/L) and Adr. The cells were spun down at 3,500 *r*/min for 7 min. The cells were then fixed in 70% ethanol for 30 min and the pellet obtained after centrifugation was dissolved in PBS. Ribonuclease A (RnaseA; sigma-R6148; 10 mg/mL) at a volume of 5 μL was added and incubated for 30 min at 37℃. Propidium iodide (PI; sigma-P4170; 10 μg/mL) was added and incubated in dark for 15 min and were filtered through 0.75 μm filter and analyzed by flow cytometry FACS Aria II (BD Biosciences). The cells were acquired and results were interpreted using cell quest pro analysis [21].

### Colony forming assay

Single-cell suspensions of “MDA-MB-231” cells were seeded in 6-well plates at a density of 150–175 cells/well. Cells were allowed to attach for 24 h and treated using concentrations (12.5 µmol/L, 25 µmol/L, 50 µmol/L) of Orm, Orm + comb and positive control/Adr for 48 h. Afterwards, media was replaced with drug-free medium and the cells were grown for approximately 7 days. On the 7th day, the cells were fixed in 10% (v/v) formalin and stained with crystal violet for 30 min. The excess stain was removed by washing with 1× PBS and plate was air-dried. To quantify the rate of colony formation, the stained cells in the form of colonies were dissolved in 10% (v/v) acetic acid and the absorbance was quantified using Varioskan Flash multimode reader (Thermo Scientific) at 540 nm [22]. Data from three independent experiments was presented as percentage of control [mean ± standard error of the mean (SEM)]. The colony forming rates were calculated using the formula.


$$\text{Colony formation rate }\left(\text{CFR}\right)=100\;\times \;\frac{\text{Mean experimental absorbance value}}{\text{Mean control absorbance value}}$$

### Chorioallantoic membrane assay

Anti-angiogenetic activity of samples were estimated following the method reported by Ribatti [23] in 2017. Zero-day old specific pathogen-free embryonated eggs were obtained from the Regional Poultry Farm, Kudappanakunnu, Thiruvananthapuram, Kerala. The eggs were horizontally placed and incubated for 3 days at 37℃ and 50% humidity in an incubator. On the 3rd day, the eggs were injected with the 20 µL of 25 µmol/L and 50 µmol/L of Orm and Orm + comb dissolved in PBS by making a pore on the upper part of the egg kept horizontally. The positive control consisted of 20 µL of Adr in PBS while the negative control comprised of 20 µL of PBS. The pores were then sealed thoroughly and incubated further for 12 days and 15 days by shaking the egg at specified intervals of time. On the 12th day and 15th day after treatment, the eggs were taken out, broke on the top without disturbing and blood vessels were scraped into a tube. The solution was sonicated for 1 min and then centrifuged for 5 min at 5,000 *r*/min. The supernatant was collected in a volume of 20 µL and made up to 5 mL using Drabkin’s reagent for the quantification of haemoglobin. The absorbance was measured at 546 nm spectrophotometrically. The density of the vasculature was recorded as the mg of haemoglobin per dl. A graph was plotted with drug concentration on X axis and quantified mg of haemoglobin per dl on the Y axis based upon the equation provided.


$$\frac{Absorbance\;of\;test}{Absorbance\;of\;control}\;\times \;15=\text{mg}/\text{dl of hemoglobin}$$

### Reverse transcription PCR and qRT-PCR

Total RNA was extracted from the 72 h treated cells using TRIzol reagent (Takara, Japan) and then transcribed into complementary DNA (cDNA) using high capacity reverse transcription kit (Thermo Scientific) with oligo deoxy-thymidine (dT) primers following the manufacturer’s protocol. Real-time PCR amplification was done using maxima SYBR Green real-time PCR master mix with fluorescein (2×) in Miniopticon real-time PCR system (Qiagen). The reactions were performed in triplicates containing 1 µL of cDNA (10 ng/μL). The gene expression was normalized to glyceraldehyde-3-phosphate dehydrogenase (*GAPDH*) as endogenous control and the relative quantity of mRNA was calculated on the basis of 2^–ΔΔCt^ method. The data was expressed as fold increase in mRNA level in treated sample with respect to the untreated control [24].

### Statistical analysis

Each data plotted represents three experiements, with each experiment in triplicate number. The data was of normal distribuition and homogeneity of variance was analysed using using one-way analysis of variance (ANOVA) analysis followed by Tukey’s honestly significant difference test or Tukey’s honestly significant difference (HSD), using Graphpad software (version 3). *P* < 0.05 was considered as statistically significant.

## Results

### Screening and development of an effective combination

**Figure 1:**
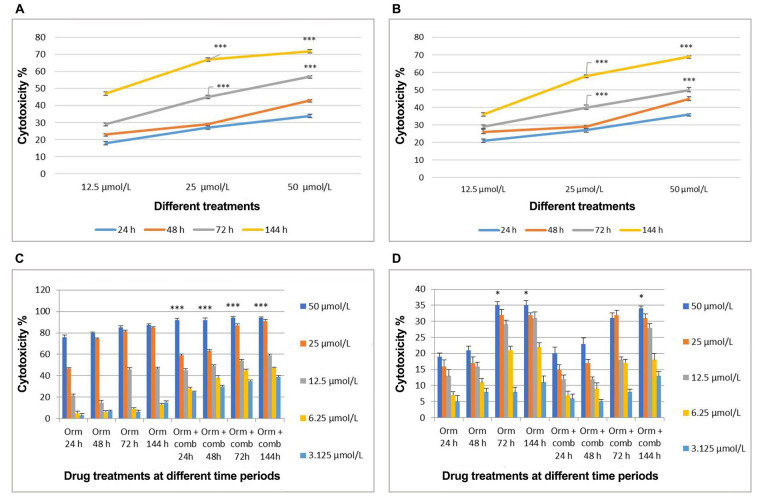
Comparative analysis of cytotoxic effects induced by treatments on “MDA-MB-231” cells and fibroblast cells over time intervals. (A) Cytotoxicity induced by sertraline at 24 h, 48 h, 72 h, and 144 h of treatment; (B) cytotoxicity induced by plumbagin at 24 h, 48 h, 72 h, and 144 h of treatment; (C) cytotoxicity induced by Orm in comparison with Orm + comb on “MDA-MB-231” cells at different time periods; (D) cytotoxicity induced by Orm in comparison with Orm + comb on fibroblast cells at different time periods. Orm: ormeloxifene; comb: sertraline and plumbagin. Data are representative of three independent experiments (mean ± SEM) and *P*-values are calculated using one-way ANOVA. ^***^ *P* ≤ 0.001 and ^*^ *P* ≤ 0.05

As single drugs always lead to intrinsic drug resistance, the possibility of synergism was explored in the present study. For this, different drugs like chlorpromazine, caffeine, topotecan hydrogen chloride (HCl), suberoylanilide hydroxamic acid (SAHA), withaferin, plumbagin, sertraline HCl, curcumin, salinomycin, carbamazepine, risperidone, amiodarone, Atovaquone, 1-amantadine, sodium valpro, montelukast, clozapine, dexamethasone and lithium carbonate were screened individually and in combination with Orm, for their cytotoxicity on “MDA-MB-231” cells by MTT assay. From this screening assay, the best combination was observed to be by combining Orm, sertraline and plumbagin which was found to be cytotoxic on “MDA-MB-231” cells. The effective combination of Orm in combination with plumbagin, a plant-based compound in ayurvedic medicine and sertraline, an antidepressant of the SSRI showed significant cytotoxicity at 25 µmol/L and 50 µmol/L concentrations, and was chosen for further studies. The cytotoxicity induced by the selected compounds sertraline and plumbagin is given as Figure 1A and 1B respectively. The cytotoxicity of Orm is given in the manuscript along with the combination as comparison.

### Cell viability assay

Cell viability assay results of combination showed cytotoxic effects than Orm alone on “MDA-MB-231” cells in a concentration and time dependent manner. In “MDA-MB-231” cells, Orm alone at 50 µmol/L concentration exerted 76%, 80%, 85% and 87% cytotoxicity when compared to the combination which exerted 92%, 92%, 94% and 94% cytotoxicity at 24 h, 48 h, 72 h and 144 h respectively. The combination exerted more cytotoxicity of 94%, 91% and 59% when compared to 87%, 85% and 47% (Orm alone) at 50 µmol/L, 25 µmol/L and 12.5 µmol/L concentrations respectively after 72 h treatment (Figure 1C). The *P* values of Orm vs. Orm+ comb groups at each concentration and time point were calculated to be highly significant.

However, the cytotoxicity displayed on normal fibroblast cells by both Orm and the combination was less than 35% at even the highest concentration of 50 µmol/L used, after 72 h treatment time (Figure 1D). The results highlight the potential of the combination than Orm when used as single drug in inducing cytotoxicity, sparing the normal cells to a large extent. The morphological analyses using fluorescent staining supports that the untreated “MDA-MB-231” cells appeared green in colour and healthy (Figure S1A) whereas the treated cells (25 µmol/L and 50 µmol/L) and Orm + comb (25 µmol/L and 50 µmol/L) appeared orange to red colour with lost morphology indicates subsequent DNA damage (Figure S1B–E).

### Cell cycle arrest induced by the compounds

**Figure 2:**
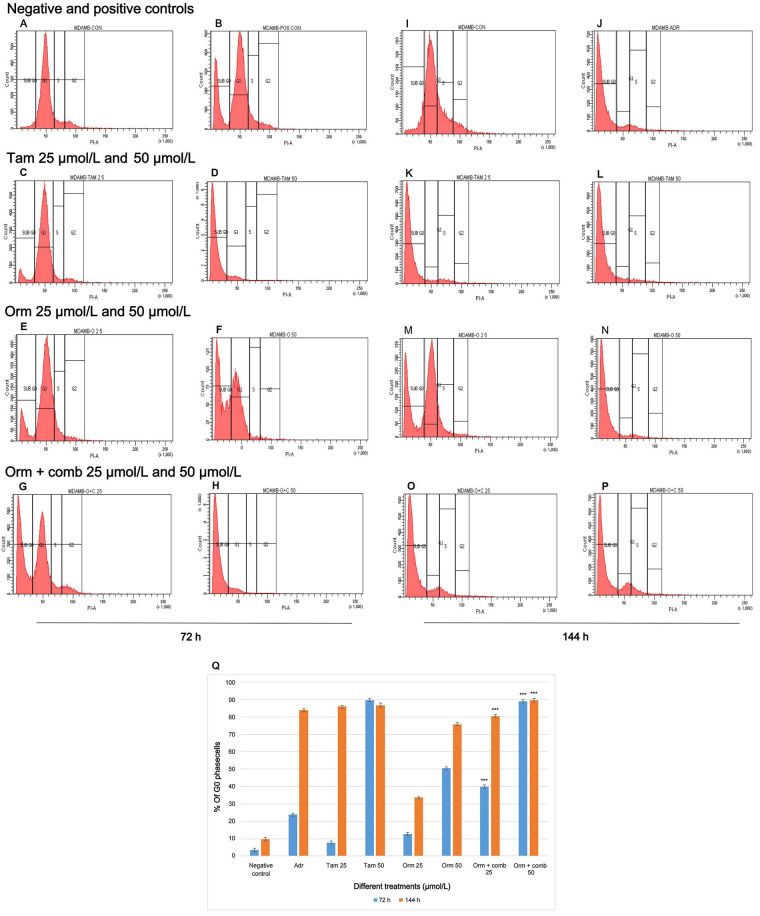
Cell Cycle Expression by Flow Cytometry: Effects of different treatments on “MDA-MB-231” cells. (A) Distribution of cells in different phases of the cell cycle in negative control—72 h; (B) distribution of cells in different phases of the cell cycle treated with positive control—72 h; (C, D) distribution of cells in different phases of the cell cycle treated with Tam 25 µmol/L and Tam 50 µmol/L—72 h; (E, F) distribution of cells in different phases of the cell cycle treated with Orm 25 µmol/L and Orm 50 µmol/L—72 h; (G, H) distribution of cells in different phases of the cell cycle treated with Orm + comb 25 µmol/L and Orm + comb 50 µmol/L—72 h; (I) distribution of cells in different phases of the cell cycle in negative control—144 h; (J) distribution of cells in different phases of the cell cycle treated with positive control—144 h; (K, L) distribution of cells in different phases of the cell cycle treated with Tam 25 µmol/L and Tam 50 µmol/L—144 h; (M, N) distribution of cells in different phases of the cell cycle treated with Orm 25 µmol/L and Orm 50 µmol/L—144 h; (O, P) distribution of cells in different phases of the cell cycle treated with Orm + comb 25 µmol/L and Orm + comb 50 µmol/L—144 h; (Q) comparison of percentage of cells in sub G0 phase of the cell cycle induced by Orm and its combination after 72 h and 144 h treatment on “MDA-MB-231” cells. Adr: adriamycin; Tam: tamoxifen; Orm: ormeloxifene; comb: sertraline and plumbagin. All values are shown as mean ± SD. The statistical analysis shows ^***^: highly significant, *P* < 0.001. MDAMB-CON: negative control; MDAMB-POS CON/MDAMB ADR: positive control/adriamycin MDAMB-TAM 25: Tam 25 µmol/L; MDAMB TAM 50: Tam 50 µmol/L; MDAMB O 25: Orm 25 µmol/L; MDAMB O 50: Orm 50 µmol/L; MDAMB O+C 25: Orm + comb 25 µmol/L; MDAMB O+C 50: Orm + comb 5 µmol/L

In “MDA-MB-231” cells, the sub G0 phase which quantifies apoptosis, increased from 3.6% (72 h) to 9.7% (144 h). For Adr, the sub G0 phase cells at 72 h were lesser (23.8%) but increased to 83.8% at 144 h. Tam (25 µmol/L) treated cells were not very effective in eliciting apoptosis in 72 h (7.8%), but significantly increased to 86% in 144 h. Tam (50 µmol/L) treated cells induced 89.7% and 86.7% cells at 72 h and 144 h respectively. Orm (25 µmol/L) treated cells did not show significant percentage of sub G0 cells. But Orm (50 µmol/L) treated cells induced 50.4% and 75.8% sub G0 phase cells at 72 h and 144 h respectively. The combination treatment (25 µmol/L) treated cells in the sub G0 phase increased from 39.8% (72 h) and to 80.3% (144 h). The combination treatment using 50 µmol/L increased sub G0 phase cells from 89% (72 h) and to 89.5% (144 h). The combination treatment (50 µmol/L) was observed to be more effective in eliciting apoptosis with the increase in sub G0 phase cells. Tam (50 µmol/L) treatment at both time points also was found to be at par with Adr treatment (Figure 2A–P). Comparative representation of the percentage of cells in sub G0 phase of the cell cycle induced by Orm and its combination after 72 h and 144 h treatment on “MDA-MB-231” cells is depicted in Figure 2Q.

### Apoptotic effects by the translocation of phosphatidyl serine

**Figure 3:**
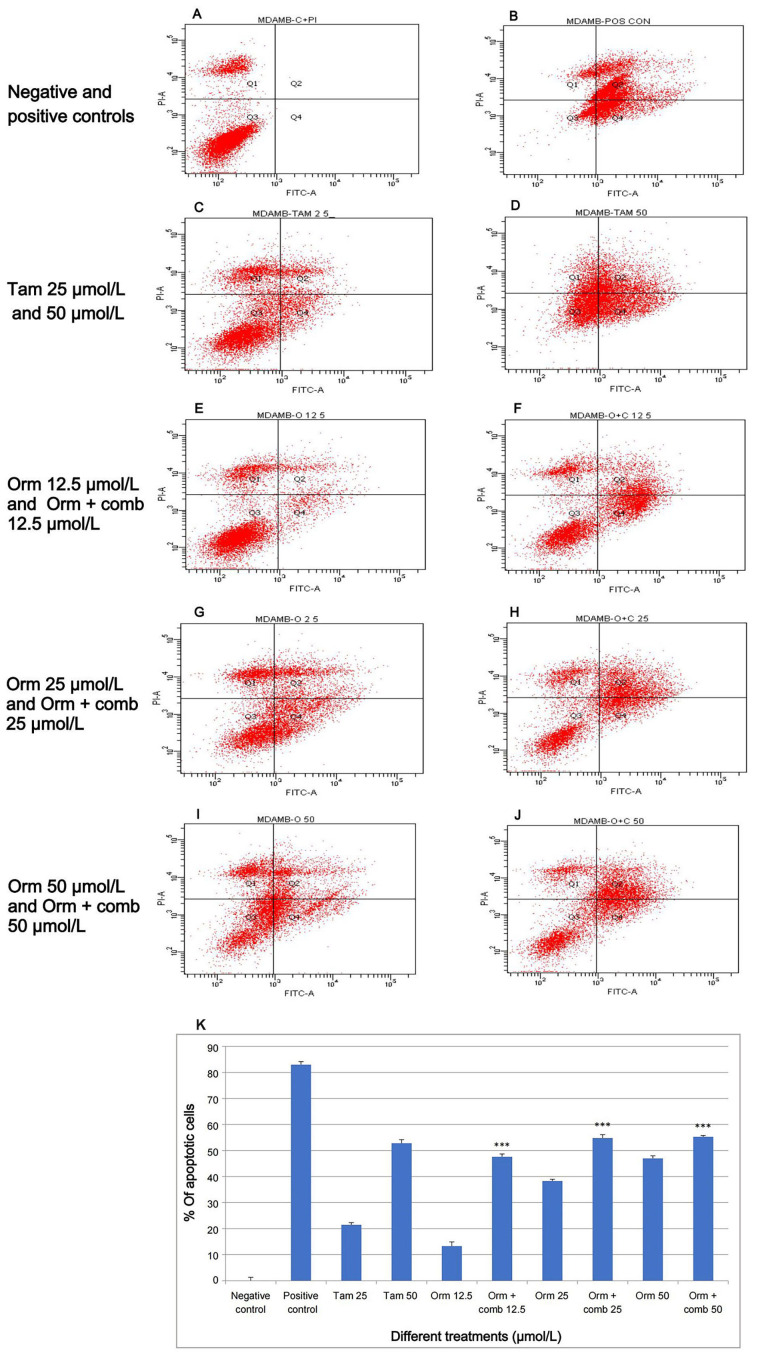
Apoptosis induction by annexin V-FITC by flow cytometry: the variations in fluorescence intensity and annexin V-FITC binding provide insights into percentage distribution of cells in different states such as viability, early apoptosis, late apoptosis and necrosis, within a given cell population after different treatments. (A) Relative proportion of cells in negative control; (B) relative proportion of cells after treatment with the positive control; (C, D) relative proportion of cells after treatment with Tam 25 µmol/L and Tam 50 µmol/L; (E, F) relative proportion of cells after treatment with Orm 12.5 µmol/L and Orm + comb 12.5 µmol/L; (G, H) relative proportion of cells after treatment with Orm 25 µmol/L and Orm + comb 25 µmol/L; (I, J) relative proportion of cells after treatment with Orm 50 µmol/L and Orm + comb 50 µmol/L; (K) graphical representation of annexin V-FITC expression by flow cytometry for detection of apoptosis on “MDA-MB-231” cells, Orm vs. combination—48 h. Orm + comb (12.5 µmol/L) vs. Orm (50 µmol/L) showed ns (*P* > 0.05) which proves that the combination even at 1/3rd concentration had an equal activity of Orm alone. Tam: tamoxifen; Orm: ormeloxifene; comb: sertraline and plumbagin. All values are shown as mean ± SD. The statistical analysis shows ^***^: highly significant, *P* < 0.001

The present study showed that Orm 50 µmol/L, 25 µmol/L and 12.5 µmol/L induced 47%, 38.3% and 13.3% of apoptotic cells whereas the combination 50 µmol/L, 25 µmol/L and 12.5 µmol/L induced 47.6%, 54.8% and 55.3% of apoptotic cells after 48 h of treatment. The combination treatment at 12.5 µmol/L vs. Orm (50 µmol/L) was ns, (*P* > 0.05). This showed that the results of both treatments were very much comparable and the combination treatment required only 1/3rd of concentrations to achieve the same result as that of Orm (50 µmol/L). Also, it was derived statistically that the combination (50 µmol/L) and (25 µmol/L) vs. Orm (50 µmol/L) was highly significant. In the experiment Figure 3A represents the negative control. Taking into account, Tam, and Adr for comparison of effects, it was observed that Tam (50 µmol/L) induced 52.8% and Adr caused 83% apoptosis (Figure 3B−J). The graphical representation in Figure 3K illustrates apoptosis detection on "MDA-MB-231" cells through annexin V-FITC expression using Orm and Orm + comb after 48 h by flow cytometry analysis.

### Active caspase-3 expression of Orm vs. combination of 72- and 144-hour comparison by flow cytometry

**Figure 4:**
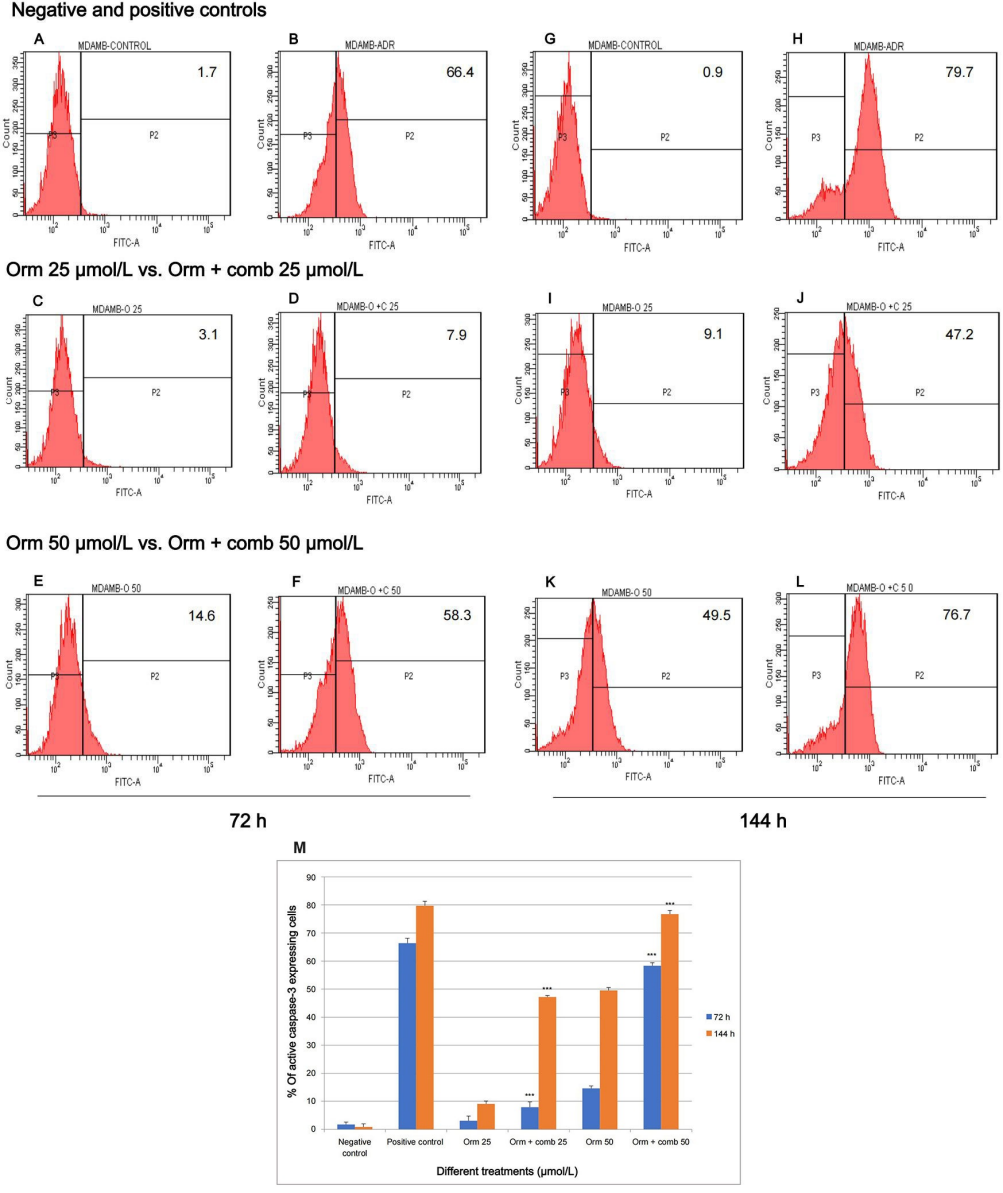
Assessment of active caspase-3 expression in “MDA-MB-231” cells: effect of Orm and Orm + comb after 72 h and 144 h of incubation. (A, B) Expression of fluorescence intensity by negative control and positive control after 72 h; (C, D) expression of fluorescence intensity by Orm 25 µmol/L and Orm + comb 25 µmol/L after 72 h; (E, F) expression of fluorescence intensity by Orm 50 µmol/L and Orm + comb 50 µmol/L after 72 h; (G, H) expression of fluorescence intensity by negative control and positive control after 144 h; (I, J) expression of fluorescence intensity by Orm 25 µmol/L and Orm + comb 25 µmol/L after 144 h; (K, L) expression of fluorescence intensity by Orm 50 µmol/L and Orm + comb 50 µmol/L after 144 h; (M) graphical representation of % of more fluorescence intensity cells expressing active caspase-3 expression induced by Orm and Orm + comb on “MDA-MB-231” cells after 72 h and 144 h of incubation. Orm: ormeloxifene; comb: sertraline and plumbagin. All values are shown as mean ± SD. The statistical analysis shows ^***^: highly significant, *P* < 0.001

The flow cytometry studies pertaining to the expression of active caspase-3 is depicted in (Figure 4). Fluorescence intensity was directly proportional to the active caspase-3 expressing cells. The study reveals that Adr induced 66.4% and 79.7% more fluorescence intensity following treatment indicating active positive caspase-3 cells expression on “MDA-MB-231” cells after 72 h and 144 h of treatment respectively. Orm (25 µmol/L) was found to induce fluorescence intensity of only 3.1% and 9.1% after 72 h and 144 h whereas combination (25 µmol/L) induced 7.9% and 47.2% intensity. Orm (50 µmol/L) induced 14.6% and 49.5% fluorescence intensity at 72 h and 144 h respectively whereas the combination at the same concentration of (50 µmol/L) induced a sharp increase in the intensity to 58.3% and 76.7% at the time points mentioned above. There was highly significant deviation in the values in treatments of Orm (50 µmol/L) vs. combination (50 µmol/L) at both 72 h and 144 h (^***^ *P* < 0.001). At the same time, it was also observed that there was no significant change in the fluorescence intensity and the percentage of active caspase-3 expressing cells of combination drug (50 µmol/L) and positive control Adr was remarkably comparable which suggested the efficacy of the combination developed (Figure 4A–J). The depiction in Figure 4M, illustrates an increase in fluorescence intensity, signifying active caspase-3 expression. This is demonstrated by calculating the percentage of “MDA-MB-231” cells expressing active caspase-3 following treatment with Orm alone and in combination at concentrations of 50 µmol/L, 25 µmol/L, and 12.5 µmol/L. The comparison is made against untreated control cells and those treated with Adr.

### Colony formation assay

**Figure 5:**
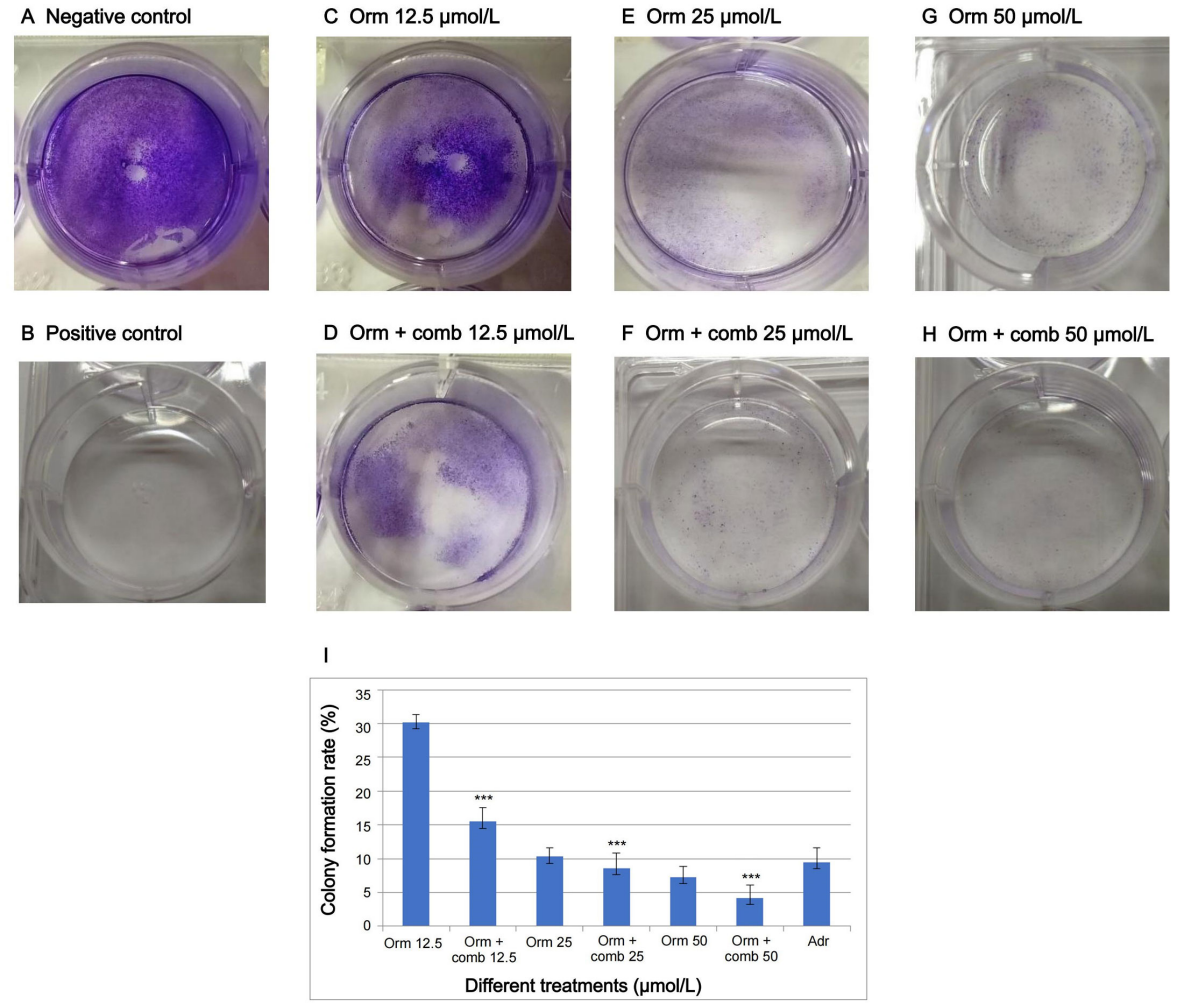
Drugs inhibit the proliferative potential of “MDA-MB-231” cells as assessed by clonogenic assay. (A, B) represents negative and positive control; (C–H) represents the clones produced in response to different treatments of Orm and Orm + comb at 50 µmol/L, 25 µmol/Land 12.5 µmol/L; (I) graphical representation of percentage of colony formed by “MDA-MB-231” triple negative breast cancer cells. Orm: ormeloxifene; comb: sertraline and plumbagin; Adr: adriamycin. All values are shown as mean ± SD. The statistical analysis shows ^***^: highly significant, *P* < 0.001

The combination drug exposure resulted in a significant reduction of clones in “MDA-MB-231” cell lines. The number of “MDA-MB-231” clones was considerably diminished at 12.5 µmol/L, 25 µmol/L and 50 µmol/L concentrations, which showed the efficacy of combination over Orm alone (Figure 5). The colony forming rate in wells with clones produced by “MDA-MB-231” cells is illustrated in Figure 5I.

### CAM assay

**Figure 6:**
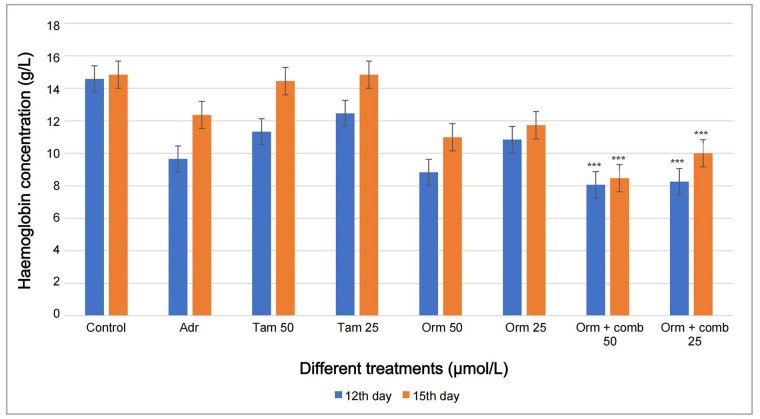
Graphical representation of the comparison of 12th day and 15th day CAM assay haemoglobin concentration by Drabkins assay for anti-angiogenesis activity. Adr: adriamycin; Tam: tamoxifen; Orm: ormeloxifene; comb: sertraline and plumbagin. All values are shown as mean ± SD. The statistical analysis shows ^***^: highly significant, *P* < 0.001

The haemoglobin (g/L) of the blood vessels formed after treatment with Orm and its combination was observed lesser when compared to the blood vessels formed by the treatment using Tam on both 12th day and 15th day. The haemoglobin content in the blood vessels treated with Orm + comb at 50 µmol/L was calculated to be highly significant (^***^ *P* < 0.05) than Tam, Adr and Orm as single drugs proving the synergistic effect of the drugs (Figure 6).

### Gene expression studies using real-time PCR

The primer information and sequences of primers used for the real-time PCR is given as Table S1.

**Figure 7:**
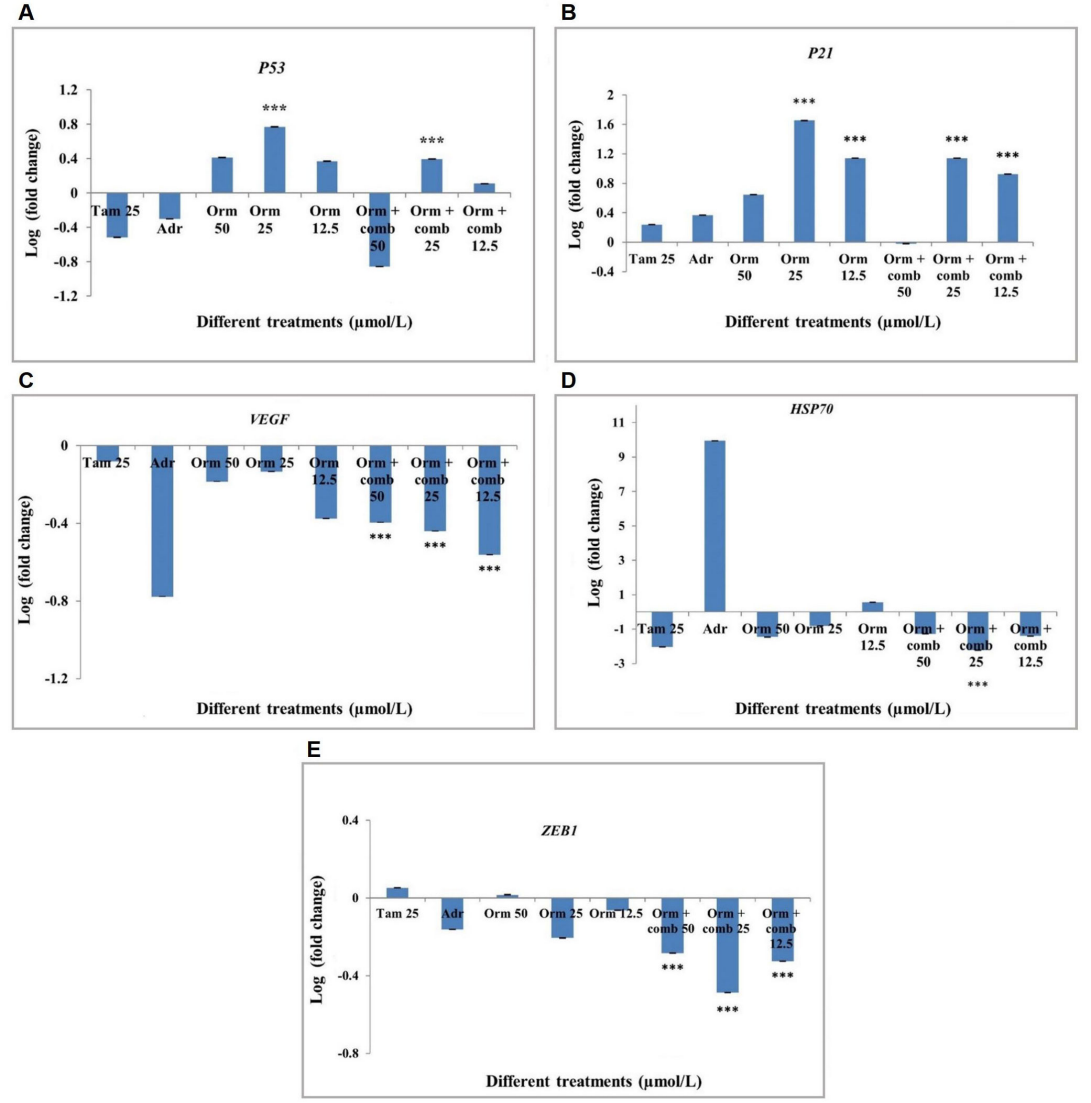
Gene expression profiling by real-time PCR in “MDA-MB-231” cells after different drug treatments. (A) *P53* expression in “MDA-MB-231” cells; (B) *P21* expression in “MDA-MB-231” cells; (C) *VEGF* expression in “MDA-MB-231” cells; (D) *HSP70* expression in “MDA-MB-231” cells; (E) *ZEB1* expression in “MDA-MB-231” cells. Adr: adriamycin; Tam: tamoxifen; Orm: ormeloxifene; comb: sertraline and plumbagin. The treatments were done with 50 µmol/L, 25 µmol/L and 12.5 µmol/L of Orm and Orm + comb and compared with Tam and Adr. The results were expressed in log fold calculated by 2^-∆∆Ct^ method, normalized to *GAPDH*. Triplicates were used in the analyses and statistically significant changes in the expression were observed for the mRNAs as shown *n* = 3, ^***^ *P* < 0.001. Error bars represent SD

The *P53* and *P21* genes was found to be upregulated significantly in both combinatorial treatment as well the Orm alone at 25 µmol/L concentration than Tam and Adr (Figure 7A and 7B). The transcriptional activation of the tumor suppressor genes is expected to suppresses the development of the tumor by growth arrest, DNA repair and apoptosis. The growth arrest will eventually stop the cell cycle progression and hence prevent the replication of the corrupted DNA.

The transcriptional activation of *VEGF* was found to be downregulated in all concentration of the drugs used. Significant downregulation of *VEGF* was observed in all treatments with Orm and combination at 25 µmol/L and 12.5 µmol/L concentrations, henceforth Orm as well as the combination drugs was observed to inhibit blood vessel formation and therefore inhibits metastatsis (Figure 7C). This combinatorial approach can be effectively used as an anti-angiogenesis agent for breast cancer therapy.

The down regulation of the Heat shock proteins in the present study is expected to slow down the central components of the cellular network of the molecular chaperones and folding catalysts so that corrupted proteins formed could not be translated. The combination of drugs at 25 µmol/L concentration was found to be significant in downregulating *HSP70* gene expression (Figure 7D).

Zinc finger E-box binding homeobox 1 (*ZEB1*) expression was down regulated following the treatment of cells with Orm + comb at all concentrations (50 µmol/L, 25 µmol/L, 12.5 µmol/L). The highest down regulation of *ZEB1* expression was shown by the Orm + comb at 25 µmol/L. Orm + comb combination at both 50 µmol/L and 12.5 µmol/L showed a comparable decrease (Figure 7E). The decrease in the *ZEB1* gene which is metastatic inducing gene is expected to decrease the metastasis following treatment. Also, the lower expression of the gene is expected to inhibit the epithelial-mesenchymal transition (EMT) which is an important hallmark in cancer metastasis.

## Discussions

Orm was reported already to be a promising SERM which can be repositioned from contraceptive purpose on fast track for the development of an efficacious anticancer drug (reported by corresponding author). The anticancer activity of Orm was compared with that of Tam and was studied through in vitro and in vivo assays [25].

The identification of novel compounds are crucial for the discovery of new lead for cancers. The statistical study states that the combinatorial approach of new compounds accounts for alternative strategies for cancer treatment mostly to combat the inherent drug resistance following some course of treatment. In the present study, humble attempt has been made for cytotoxic screening of the Orm with combination to correlate their anticancer activities using different methodologies. The apoptotic effects of the drugs were extensively studied as defective apoptosis is reported to be a major causative factor in the development and progression of cancer. Activation of apoptosis is the key molecular mechanism responsible for the anti-cancer activities of most of the currently studied potential anti-cancer agents.

In the present study, viability assays using Orm in combination demonstrated greater efficacy in killing cancer cells than Orm used as a single drug on “MDA-MB-231” cell lines. The significant activation of apoptosis using the combinatorial approach could be explored and tapped to develop the combination as potential combo-candidate for treating breast cancer in the future. The induction of apoptosis by the drugs were studied by observing morphological features like shrinkage of cells which in turn leads to rounding off the cells and loss of integrity. Compounds treated with Orm and combination at different concentrations was observed to cause reduction in proliferation which subsequently caused cell death.

Mode of apoptosis in the cell death induced by the combinatorial approach was confirmed using AO-EB dual staining by visualizing the internal changes induced by the compounds. AO was permeable to both viable and non-viable cells while EB was taken up only by damaged and dead cells, whose membrane integrity was lost. Live cells would appear as green and apoptotic cells as orange in color in which all the three compounds showed morphological and internal changes. Hence it was confirmed that the cytotoxicity induced by the three compounds ultimately induced apoptotic signals eventually leading to cell death.

The nature of cell death was further confirmed to be apoptosis by double labelling techniques using fluorescent labelled annexin V/PI which distinguished apoptotic and necrotic cells. The cells were impermeable to PI during early stage of apoptosis whereas permeable to both annexin FITC and PI dyes at a later stage of apoptosis. Induction of apoptosis was confirmed and quantified by annexin V-FITC using flow cytometry following 48 h treatment. The combinatorial approach had a pronounced effect in causing apoptosis. Colony forming assay supported the effect of the drugs by decreasing the colony formation and significant reduction in angiogenesis was confirmed by CAM assay. The genes for real-time PCR were selected due to the following strategic functions in cancer cells. *P53* gets activated in the response to DNA damage, acting as a classic transcription factor, *P53* induces the expression of *P21*, which in turn inhibits cyclin-dependent kinases (CDKs), resulting in cell-cycle arrest [26]. *ZEB1* is a pleiotropic transcription factor frequently expressed in carcinomas. *ZEB1* orchestrates the transcription of genes in the control of several key developmental processes and tumor metastasis via the EMT [27]. Heat shock proteins profoundly impact malignant progression across multiple cancer types by manipulating cancer hallmarks including anti-apoptosis, proliferation, metastasis, and angiogenesis [28–31].

The anti-angiogenic property of the drug was further confirmed by the Gene expression studies of *VEGF* which provided an effective down-regulation of the gene responsible for the production of Proteins associated with the angiogenesis. Similarly, the down-regulation of *ZEB1* and *HSP70* is highly significant in case of combinatorial approach compared to the use of Orm alone. The *P53* and *P21* genes exhibited significant upregulation in response to treatment with Orm with combination. The gene expression studies revealed transcriptional activation of *P21* and *P53*, often referred to as the guardians of the genome, thus protecting the cells for DNA repair. Changes in the transcription of key genes block the cell cycle, angiogenesis and metastasis by promoting apoptosis and differentiation. Hence the combination of sertraline and plumbagin along with Orm serves as an effective potent novel combinatorial approach in bringing down the aggressiveness of the breast cancer. More in vivo and other in vitro mechanistic studies are warranted to prove the efficacy of the combination treatment.

It can be concluded from the results obtained in the present study that the combinatorial approach using sertraline, plumbagin and Orm possess effective anticancer activity in breast cancer comparable or even better than Tam which is clinically used, at the same time with lesser cytotoxicity on normal cells. Comparatively, the combination worked effectively as a better candidate and found to induce apoptosis than Orm alone. The changes in the transcriptional activity of the genes selected were highly significant showing anti-cancer mechanisms of the Orm combination. Further studies are warranted to develop the combinatorial approach for treatment of breast cancer and to address the capability of the combination to combat inherent resistance mechanisms following treatment.

## Abbreviations

AO: acridine orange
CAM: chorioallantoic membrane
EB: ethidium bromide
HSP70: 70 kDa heat shock protein
PI: propidium iodide
SD: standard deviation
SEM: standard error of the mean
SERM: selective estrogen receptor modulator
VEGF: vascular endothelial growth factor
ZEB1: zinc finger E-box binding homeobox 1

## Supplementary materials

The supplementary material for this article is available at: https://www.explorationpub.com/uploads/Article/file/1002235_sup_1.pdf.

## Declarations

### Availability of data and materials

The main data are included in this manuscript. All data are available from the corresponding author on reasonable request. Email: lakshmis@lifecarehll.com.

### Copyright

© The Author(s) 2024.
