# Supplementary material for: Evaluation of molecular effects associated with apoptosis, tumour progression, angiogenesis and metastasis by a novel combination of drugs with ormeloxifene in triple negative breast cancer cells
Source: Explor Target Antitumor Ther. 2024 Jun 11;5(3):551–67. doi: 10.37349/etat.2024.00235 (PMC11220290; doi:10.37349/etat.2024.00235)
Supplement: Supplementary file 1 [file 1002235_sup_1.pdf]

Supplementary materials

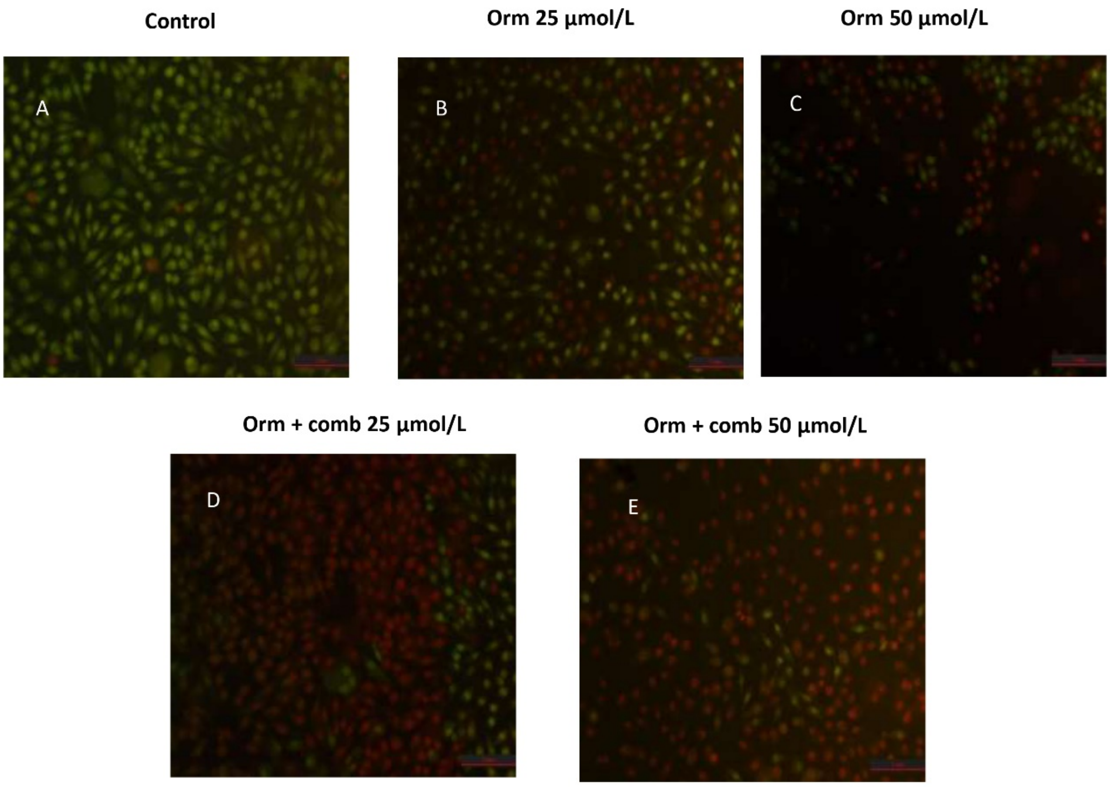

Figure S1. Nuclear morphological analysis by AO/EB staining. (A) Control appeared in green colour with uniform distribution; (B) Orm 25 µmol/L which allowed EB to enter and stain the nuclei orange; (C) Orm 50 µmol/L: showed decreased cell number; (D) Orm + comb 25 µmol/L: the cells showed orange fluorescence by EB staining effectively; (E) Orm + comb 50 µmol/L appeared orange indicates subsequent DNA damage

Primer information

The primers were designed using the online primer designing software, Primer3 (https://primer3.ut.ee/). Sequances downloaded in FASTA format files used along with subsequent parameters settings. Primer length: 20–26 bases; primer melting temperature around 60 to 64°C; and product size range: <250 bp for heat-shock proteins 70 (Hsp-70), Zinc finger E box binding Homeobox 1 (Zeb1), Vascular Endothelial Growth Factor (VEGF), Tumor Suppressor Protein (p53), Cyclin-dependent kinase inhibitor (p21), Glyceraldehyde 3-phosphate dehydrogenase (GAPDH).

Table S1: Sequences of primers used for the real-time PCR

**Primer**	**Sequence**	**Accession number**
ZEB-1 (Forward)<br>*ZEB-1* (Reverse)	TGCACTGAGTGTGGAAAAGC<br>TGGTGATGCTGAAAGAGACG	NM_001128128.3
VEGF (Forward)<br>*VEGF* (Reverse)	ACGGTCCCTCTTGGAATTGG<br>GGCCGCGGTGTGTCTA	M32977.1
P53 (Forward)<br>*P53* (Reverse)	TGTGTCTGAGGGGTGAACG<br>GGACGGTTGGTTCCTGAGTTA	U94788.1
P21(Forward)<br>*P21*(Reverse)	TCAGTTCCTTGTGGAGCCG<br>AGTCGAAGTTCCATCGCTCA	NM_001374511.1
HSP70 (Forward)<br>*HSP70* (Reverse)	TCCAGGAATCTGAAGAACGACC<br>GGTGTGACTTCCATCCACTC	L12723.2
GAPDH (Forward)<br>*GAPDH* (Reverse)	GGCAAATTCCATGGCACCGT<br>GCATCGCCCCACTTGATTTT	NM_001256799.3

We used the following accession number sequence for the primer design and manually sequences of ZEB-1 (NM_001128128.3), VEGF (M32977.1), P53 (U94788.1), P21 (NM_001374511.1), HSP70 (L12723.2) & GAPDH (NM_001256799.3) were copy-pasted in Word document and checked each forward and reverse primer sequence alignment as shown in the word document attached in this e-mail. We used reverse complement sequences for reverse primer verification.

**Primer**	**Sequence**	**Accession number**
ZEB-1 (Forward)<br>*ZEB-1* (Reverse)	TGCACTGAGTGTGGAAAAGC<br>TGGTGATGCTGAAAGAGACG	NM_001128128.3
ZEB-1 Reverse compliment for reverse primer:	CGTCTCTTTCAGCATCACCA	
VEGF (Forward)<br>*VEGF* (Reverse)	ACGGTCCCTCTTGGAATTGG<br>GGCCGCGGTGTGTCTA	M32977.1
VEGF Reverse compliment for reverse primer:	TAGACACACCGCGGCC	

P53 (Forward)<br>*P53* (Reverse)	TGTGTCTGAGGGGTGAACG<br>GGACGGTTGGTTCCTGAGTTA	U94788.1
P53 Reverse compliment for reverse primer:	TAACTCAGGAACCAACCGTCC	
P21(Forward)<br>*P21*(Reverse)	TCAGTTCCTTGTGGAGCCG<br>AGTCGAAGTTCCATCGCTCA	NM_001374511.1
P21 Reverse compliment for reverse primer:	TGAGCGATGGAACTTCGACT	
HSP70 (Forward)<br>*HSP70* (Reverse)	TCCAGGAATCTGAAGAACGACC<br>GGTGTGACTTCCATCCACTC	L12723.2
HSP70 Reverse compliment for reverse primer:	GAGTGGATGGAAGTCACACC	
GAPDH (Forward)<br>*GAPDH* (Reverse)	GGCAAATTCCATGGCACCGT<br>GCATCGCCCCACTTGATTTT	NM_001256799.3
GAPDH Reverse compliment for reverse primer:	AAAATCAAGTGGGGCGATGC	

In addition, we have re-verified primer-blast software (link given in your attached Word file). We changed the setting as highlighted in below screenshot of database to **nr after that only sequences are given in the forward and reverse primer box and the results were found to be sequences compatible with VEGF (M32977.1), P53 (U94788.1) & HSP70 (L12723.2) as shown in subsequent screenshots.**

Settings change in the primer BLAST software

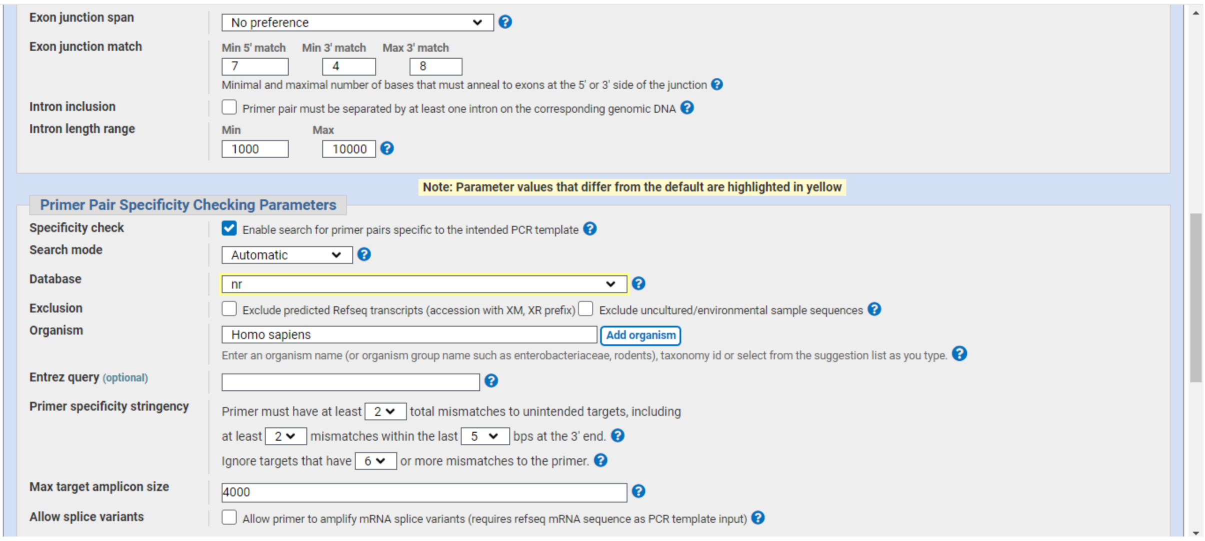

VEGF

Primer-BLAST » JOB ID:0tgMkA6KAylkGJMdnn23L-Rmph3Jdb0AyA

Primer-BLAST Results

Input PCR template none
Specificity of primers Target templates were found in selected database: Nucleotide collection (nt) (Organism limited to Homo sapiens)
Other reports ▹Search Summary

— Detailed primer reports

Primer pair 1

	Sequence (5'->3')	Length	Tm	GC%	Self complementarity	Self 3' complementarity
Forward primer	ACGGTCCCTCTTGGAATTGG	20	59.67	55.00	4.00	2.00
Reverse primer	GGCCGCGGTGTGTCTA	16	58.93	68.75	6.00	2.00

Products on target templates
>M32977.1 Human heparin-binding vascular endothelial growth factor (VEGF) mRNA, complete cds

```
product length = 111
Forward primer  1     ACGGTCCCTCTTGGAATTGG  20
Template        868   ....................  887

Reverse primer  1     GGCCGCGGTGTGTCTA  16
Template        978   ................  963
```

TP53

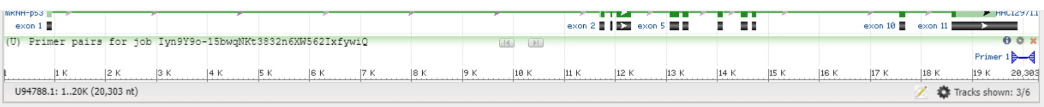

— Detailed primer reports

Primer pair 1

	Sequence (5'->3')	Template strand	Length	Start	Stop	Tm	GC%	Self complementarity	Self 3' complementarity
Forward primer	TGTGTCTGAGGGGTGAACG	Plus	19	19881	19899	59.25	57.89	3.00	2.00
Reverse primer	GGACGGTTGGTTCCTGAGTTA	Minus	21	20106	20086	59.65	52.38	3.00	2.00
Product length	226								

Products on intended targets
>U94788.1 Human p53 (TP53) gene, complete cds

```
product length = 226
Forward primer  1       TGTGTCTGAGGGGTGAACG  19
Template        19881   ...................  19899

Reverse primer  1       GGACGGTTGGTTCCTGAGTTA  21
Template        20106   .....................  20086
```

Products on potentially unintended templates
>CP139526.1 Homo sapiens isolate NA24385 chromosome 17

HSP70

Primer-BLAST » JOB ID:8PousAQzCZsupQygAcAoknvbOaBWyCK9Vw

Primer-BLAST Results

Input PCR template none
Specificity of primers Target templates were found in selected database: Nucleotide collection (nt) (Organism limited to Homo sapiens)
Other reports ▹Search Summary

— Detailed primer reports

Primer pair 1

	Sequence (5'->3')	Length	Tm	GC%	Self complementarity	Self 3' complementarity
Forward primer	TCCAGGAATCTGAAGAACGACC	22	59.77	50.00	5.00	0.00
Reverse primer	GGTGTGACTTCCATCCACTC	20	57.90	55.00	3.00	1.00

Products on target templates
>L12723.2 Homo sapiens heat shock protein 70 (hsp70) mRNA, complete cds

```
product length = 191
Forward primer  1      TCCAGGAATCTGAAGAACGACC  22
Template        2140   ......................  2161

Reverse primer  1      GGTGTGACTTCCATCCACTC  20
Template        2330   ....................  2311
```

Human heparin-binding vascular endothelial growth factor (VEGF) mRNA, complete cds

GenBank: M32977.1

GenBank Graphics

>M32977.1 Human heparin-binding vascular endothelial growth factor (VEGF) mRNA, complete cds

CAGTGTGCTGGCGGCCCGGCGCGAGCCGGCCCGGCCCCGGTCGGGCCTCCGAAACCATGAACTTT
CTGCT
GTCTTGGGTGCATTGGAGCCTCGCCTTGCTGCTCTACCTCCACCATGCCAAGTGGTCCCAGGCTGC
ACCC
ATGGCAGAAGGAGGAGGGCAGAATCATCACGAAGTGGTGAAGTTCATGGATGTCTATCAGCGCAG
CTACT
GCCATCCAATCGAGACCCTGGTGGACATCTTCCAGGAGTACCCTGATGAGATCGAGTACATCTTCA
AGCC
ATCCTGTGTGCCCCTGATGCGATGCGGGGGCTGCTGCAATGACGAGGGCCTGGAGTGTGTGCCCAC
TGAG
GAGTCCAACATCACCATGCAGATTATGCGGATCAAACCTCACCAAGGCCAGCACATAGGAGAGAT
GAGCT
TCCTACAGCACAACAAATGTGAATGCAGACCAAAGAAAGATAGAGCAAGACAAGAAAATCCCTG
TGGGCC
TTGCTCAGAGCGGAGAAAGCATTTGTTTGTACAAGATCCGCAGACGTGTAAATGTTCCTGCAAAAA
CACA
GACTCGCGTTGCAAGGCGAGGCAGCTTGAGTTAAACGAACGTACTTGCAGATGTGACAAGCCGAG
GCGGT
GAGCCGGGCAGGAGGAAGGAGCCTCCCTCAGGGTTTCGGGAACCAGATCTCTCACCAGGAAAGAC
TGATA
CAGAACGATCGATACAGAAACCACGCTGCCGCCACCACACCATCACCATCGACAGAACAGTCCTT
AATCC
AGAAACCTGAAATGAAGGAAGAGGAGACTCTGCGCAGAGCACTTTGGGTCCGGAGGGCGAGACT
CCGGCG
GAAGCATTCCCGGGCGGGTGACCCAGCACGGTCCCTCTTGGAATTGGATTCGCCATTTTATTTTTCT
TGC
TGCTAAATCACCGAGCCCGGAAGATTAGAGAGTTTTATTTCTGGGATTCCTGTAGACACACCGCGG
CCGC
CAGCACACTG

VEGF (Forward)<br>*VEGF* (Reverse)	ACGGTCCCTCTTGGAATTGG<br>GGCCGCGGTGTGTCTA
Reverse compliment for reverse primer:	TAGACACACCGCGGCC

Human p53 (TP53) gene, complete cds

GenBank: U94788.1

GenBank Graphics

>U94788.1 Human p53 (TP53) gene, complete cds

TTCCCATCAAGCCCTAGGGCTCCTCGTGGCTGCTGGGAGTTGTAGTCTGAACGCTTCTATCTTGGCG
AGA
AGCGCCTACGCTCCCCCTACCGAGTCCCGCGGTAATTCTTAAAGCACCTGCACCGCCCCCCCGCCG
CCTG
CAGAGGGCGCAGCAGGTCTTGCACCTCTTCTGCATCTCATTCTCCAGGCTTCAGACCTGTCTCCCTC
ATT
CAAAAAATATTTATTATCGAGCTCTTACTTGCTACCCAGCACTGATATAGGCACTCAGGAATACAA
CAAT
GAATAAGATAGTAGAAAAATTCTATATCCTCATAAGGCTTACGTTTCCATGTACTGAAAGCAATGA
ACAA
ATAAATCTTATCAGAGTGATAAGGGTTGTGAAGGAGATTAAATAAGATGGTGTGATATAAAGTAT
CTGGG
AGAAAACGTTAGGGTGTGATATTACGGAAAGCCTTCCTAAAAAATGACATTTTAACTGATGAGAA
GAAAG
GATCCAGCTGAGAGCAAACGCAAAAGCTTTCTTCCTTCCACCCTTCATATTTGACACAATGCAGGA
TTCC
TCCAAAATGATTTCCACCAATTCTGCCCTCACAGCTCTGGCTTGCAGAATTTTCCACCCCAAAATGT
TAG
TATCTACGGCACCAGGTCGGCGAGAATCCTGACTCTGCACCCTCCTCCCCAACTCCATTTCCTTTGC
TTC
CTCCGGCAGGCGGATTACTTGCCCTTACTTGTCATGGCGACTGTCCAGCTTTGTGCCAGGAGCCTC
GCAG
GGGTTGATGGGATTGGGGTTTTCCCCTCCCATGTGCTCAAGACTGGCGCTAAAAGTTTTGAGCTTC
TCAA
AAGTCTAGAGCCACCGTCCAGGGAGCAGGTAGCTGCTGGGCTCCGGGGACACTTTGCGTTCGGGC
TGGGA
GCGTGCTTTCCACGACGGTGACACGCTTCCCTGGATTGGGTAAGCTCCTGACTGAACTTGATGAGT
CCTC
TCTGAGTCACGGGCTCTCGGCTCCGTGTATTTTCAGCTCGGGAAAATCGCTGGGGCTGGGGGTGGG
GCAG
TGGGGACTTAGCGAGTTTGGGGGTGAGTGGGATGGAAGCTTGGCTAGAGGGATCATCATAGGAGT
TGCAT
TGTTGGGAGACCTGGGTGTAGATGATGGGGATGTTAGGACCATCCGAACTCAAAGTTGAACGCCT
AGGCA
GAGGAGTGGAGCTTTGGGGAACCTTGAGCCGGCCTAAAGCGTACTTCTTTGCACATCCACCCGGTG
CTGG
GCGTAGGGAATCCCTGAAATAAAAGATGCACAAAGCATTGAGGTCTGAGACTTTTGGATCTCGAA
ACATT
GAGAACTCATAGCTGTATATTTTAGAGCCCATGGCATCCTAGTGAAAACTGGGGCTCCATTCCGAA
ATGA

TCATTTGGGGGTGATCCGGGGAGCCCAAGCTGCTAAGGTCCCACAACTTCCGGACCTTTGTCCTTC
CTGG

AGCGATCTTTCCAGGCAGCCCCCGGCTCCGCTAGATGGAGAAAATCCAATTGAAGGCTGTCAGTC
GTGGA

AGTGAGAAGTGCTAAACCAGGGGTTTGCCCGCCAGGCCGAGGAGGACCGTCGCAATCTGAGAGGC
CCGGC

AGCCCTGTTATTGTTTGGCTCCACATTTACATTTCTGCCTCTTGCAGCAGCATTTCCGGTTTCTTTTT
GC

CGGAGCAGCTCACTATTCACCCGATGAGAGGGGAGGAGAGAGAGAGAAAATGTCCTTTAGGCCGG
TTCCT

CTTACTTGGCAGAGGGAGGCTGCTATTCTCCGCCTGCATTTCTTTTTCTGGATTACTTAGTTATGGC
CTT

TGCAAAGGCAGGGGTATTTGTTTTGATGCAAACCTCAATCCCTCCCCTTCTTTGAATGGTGTGCCCC
ACC

CCCCGGGTCGCCTGCAACCTAGGCGGACGCTACCATGGCGTAGACAGGGAGGGAAAGAAGTGTGC
AGAAG

GCAAGCCCGGAGGCACTTTCAAGAATGAGCATATCTCATCTTCCCGGAGAAAAAAAAAAAAGAAT
GGTAC

GTCTGAGAATGAAATTTTGAAAGAGTGCAATGATGGGTCGTTTGATAATTTGTCGGGAAAAACAA
TCTAC

CTGTTATCTAGCTTTGGGCTAGGCCATTCCAGTTCCAGACGCAGGCTGAACGTCGTGAAGCGGAAG
GGGC

GGGCCCGCAGGCGTCCGTGTGGTCCTCCGTGCAGCCCTCGGCCCGAGCCGGTTCTTCCTGGTAGGA
GGCG

GAACTCGAATTCATTTCTCCCGCTGCCCCATCTCTTAGCTCGCGGTTGTTTCATTCCGCAGTTTCTTC
CC

ATGCACCTGCCGCGTACCGGCCACTTTGTGCCGTACTTACGTCATCTTTTTCCTAAATCGAGGTGGC
ATT

TACACACAGCGCCAGTGCACACAGCAAGTGCACAGGAAGATGAGTTTTGGCCCCTAACCGCTCCG
TGATG

CCTACCAAGTCACAGACCCTTTTCATCGTCCCAGAAACGTTTCATCACGTCTCTTCCCAGTCGATTC
CCG

ACCCCACCTTTATTTTGATCTCCATAACCATTTTGCCTGTTGGAGAACTTCATATAGAATGGAATCA
GGA

TGGGCGCTGTGGCTCACGCCTGCACTTTGGCTCACGCCTGCACTTTGGGAGGCCGAGGCGGGCGGA
TTAC

TTGAGGATAGGAGTTCCAGACCAGCGTGGCCAACGTGGTGAATCCCCGTCTCTACTAAAAAATAC
AAAAA

TTAGCTGGGCGTGGTGGGTGCCTGTAATCCCAGCTATTCGGGAGGGTGAGGCAGGAGAATCGCTT
GAACC

CGGGAGGCAGAGGTTGCAGTGAGCCAAGATCGTGCCACTACACTCCAGCCTGGGCGACAAGAACG
AAACT

CCGTCTCAAAAAAAAGGGGGGAATCATACATTATGTGCTCATTTTTGTCGGGCTTCTGTCCTTCAA
TGTA

CTGTCTGACATTCGTTCATGTTGTATATATCAGTATTTTGCTCCTTTTCATTTAGTATAGTCCATCGA
TT

GTATATCCGTCCTTTTGATGGCCTTTTGAGTTGTTTCCCATTTGCGGTTATGAAATAAAGCTGCTAT
AAA

CATTCTTGTACAATTCTTTTTGTGATCATATGTTTTCGTGTTTCTTGGAGAAATACTTAGGAGGGGA
ATT

GTGGAGGAAGTAAAAAGTAGCTGTATTTTGAACTTTTTCAGAAGCTCTGAGTTTTCCAGAGCGGTT
GTAC

CATTTTACACTCCAACTAGCAAGGTATGGGAGTTATTATGGTTGTGCCACAGCCTTCCGGACATTA
GGTA

TGTCAGTCTTTCTAATGTGGTATATCCTTGTGGTTGTAATTTACAGTTCTCTATTGACTAAGGATGT
TCA

GCATTTTTTCATGTGCCTATTGGCCATTCGTATTTTGTTTGTAAAGTAGCTCTTCGAGTCTTTTACCT
GT

TATTTTGGTTTTTTTGTTTGTTTTTATTGTTCAGTTGTGGGACTGCTTTATACTTTCTGGATACAAGT
CC

TTATCAGATCCATGAGTCGTGAATGTTTTCTTCTGATCTGTTGCGGGCCTATTTGTTTGCTTTACAG
AGT

TTACAGAATCTTAAGAGGAGTGGATTAATCTTTTTTATGTTCAGTATTTGCCTTGTCCTGTTTAGGA
CAT

CTTTTTTTTTTTTTTTAACCCCAGGGTCATGAAGATATAATCTTACATTTTCTTTTAGGACCTTTATG
GT

GGTAAGTTTTACAGTAAGGTCCTTAAGCCATTAATTAATTCTTAAAATTAATTGTTTATGGTGTGAG
GTG

TAGGAGTCAGTCTCTGGTATCTTTCCTGTATGGAAATCCAGTTATTCTGTCTCCACTTGTTGAAATA
GGC

TTCCTTTCTCTACTGAATGCTTTTAATTTTAATTATTTTACAGTTGGAGTATAGGGCTACCATTTTAG
TG

CTATTTTCTTTTTTTCTTTGTTAATTTTTGAGACAGGGACTCACACTGTTGCCCAGGCTAGAGTACA
ATG

GCACAATCAAGGCTTACTGCAGCCTCGAACCCCTGGGCTCAAGCAGTCCTCTAGCAGCCTCACGAG
TAGC

TGGGATTACTCCACCACACCCAGCTAACTATTTTATTTTTTTGTATTGACAGGATCTCACTATGTTG
CCC

AGGCTGGTCTCAAACTGCTGGCCTCAAGCTTTCATCCCATCTCGGCCTCCCAAAGTGCTGGGATTA
CAGG

TGTGAGCCACCATGCCTGACCTCTTAGTGCTATTTTCTATTTATCTCCTCTGTTCTCTGCTCTCTTTA
AA

CGTTGGAGGAAGAAACAGTACCCATCTTACACAAACTCTTCAGAAAACAGAGGAACAGACTGGGC
GCGGT

GGCTCATACCTGTAATCTCAGCACTTTGGTACGCTGAGGCAGGGGATCATTTGAGGTCGGGAGTTC
GAGA

CCAGCCTGGCCAACACGGCGAAACCCCATCTCTACTAAAATACAAAAGTAGCTAGGCGTGCACCA
TACCT

GTAATGCCAGTTACTCAGGAGGCTGAGGCACAAGAATCCCTTGAACCTGGGAAGCGGAGGTTGCA
GTGAG
CCGAGATTGCGCCACTGCACTCCAGCCTGGGCAACAGAGTGAGACCCTGTCTCAGAAAAAAAAAG
AAAGA
AAGAAAAAATAGAGGAATATTTCCCAACTTGTTTTCGAAGCCAGGATAATCCTGGTACCAAAACC
AAACA
AGGACATTATAAGAAAAGAAAATATAGACCAATATTCCTGTTAGCATAGACATGCAACAGCTAAC
CAATT
TTAGCAAACCAAACCTGGTAATATAGAAAAAAGGATAAATAGGCCAGTCGCGGTGGCTCACGCCT
GTAAT
CCCAGCACTTTGGGAGGCTGAGGCAGGCAGATCACTTGAGGTCAGGAGTTTGAGACCAGCCTGAC
CAACA
TGGTGAAACCCCGTTTCTAATAAAAATACAAAAATCAGGCTGGGCACGGTGGCTCACGCCTGTAA
TCCCA
GCACTTTGGGAGGCCGAGGTGGGCAGATCACGAGGTCAGGAGTTCAAGACCAGCCTGACCAATGT
GGTGA
AACGCCATCTCTACTAAAAATACGAAAATCAGCCGGTGTGGTGGCACCTGCCTGTAATCCCAGCTA
CTCA
GGAGGCTGAGGCAGAATTGCTTGAACCCGGGAGGCAGAGGTTGCAGTGAGCCAAGATCGTGCCAC
TGCAC
TCCAGCCTGGGCGACAGAGCAAGACTTCATCTCAAAAAAAAAAAAAATTAGCTGGGCATGGTGGT
GGGCA
CCTGAAATCCCAGCTACTCGGGAGTCTGAGGCAGGAGAATCGCTTGAACCCAGGAGGCAGAAGTT
GCACT
GAGCTGGGATCACACCATTGCACTCCAGCCTGGGCAACAGAGTGAGACTCCATCTCAAAAAAAGA
AAAAG
AAAAAGGATAAATACATTCTAACCAAATAATGTTTATCTCATGATTGTAGCTGATTCAACATTCAA
AAAT
TGGCCTGGTGCAGTAGCTCAGGCCTGTAATCCCAACATTTTAGGAGGCTGAGGCAGGAAGATCTCT
TGAG
CCCAGGATTTCAAGACCAGCCTGGGCAACATAGTCAGACTGGTCTTTACTGGGGGGAAAAAAATC
AGTCT
GTGTAATTCACCACATTAACAAAGGGAAACATAAAAACCCTATGATCATTTCAACAGATGTAGCA
AAAGC
AGTTAATGATATCAACACATATGCATGATTACAAACCAACCAACCTCCTAGCAAACTAGGGAAAG
GAAAC
TTAACTAGTTTGATAACAGGGCGTCCACAGTCGGAGTTCCACTAGCAGCATACATAATGGTAGAA
AACTC
AGTGCTGCTGGGGGCGGTGGCTCACGCCTGTAATGCCAGCGCTTTGGGAGGCCTAGGCGGGCGGA
TCACG
AGGTCAGGAGATCGAGACTGTCCTGACTAGCATGCTGAAACCCCGTCTCTACTAAAAATACAAAA
ACAAA
AAATTAGCCGGGCATGGTGGCGGGCGCCTATAGTGCCAGCTACTCGGGAGGCTGAGGCGAGAGAA
TGGCG

TGAACCCGGGAGGCGGAGCTTGCAGAGCCTAGATCGTGCCACTGCACTCCAGCCTGGGTGACAGA
GTGAG
ACTTCGTCTCAAAAAAAAAAAAAAAAAAAAAAGAAAAGAAAACTCAACGCTTTTTCCTCTAAGAT
CAGGA
ACTAGAAAAGGATTTGACTCTCACAACGTTGATACCATACTGGAGGTTTTAACCAGGCAAGAAAA
AGAAA
TAATGAGGGCCGGGTGCGGTGGCTCAGGCCTGTAATCCCAGCACTTTGGGAAGCCGAGACGGGTG
GATCA
CGAGGTCAGGAGATCGAGCCATCCTGGCTAACACGGTGAAACCCTGTCTCTACTAAATATACAAA
AAATT
AGCCGGGCGTGGTGGCGGGCGCCTGTAGTCCCAGCTACTCGGGAGGCTGAGGCAGGAGAATGGCG
TGAAC
TCAGGGGGCGGAGCTTGCAGTGAGCTGAGATCGAGCCACTGCACTCCAGCCTGGGCGACAGAGCA
AGACT
GTGTCTCAAAAAAAAAAAAAGAAAAAGAAATAATGATTAGTGGCCCGATGTCTCACGCCAGTAAT
CCCAG
CACTTTGGGAGGCCGAGGTGGGCAGATCACCTGAGGTCTGGAGTTGGAGACCAGCCTGACAAAGA
TGGTG
AAACCTCGTCTCTATTAAAATATTAAAAAAATAGCCAGGCGTTGGCCGGGTACAGTGGCTCATGCC
TGTA
ACCCCAGCACTTTGGGAGGCCGAGGTGGGTGGATCACCTGAGGTCAGGAGTTCAACACCAGCCTG
GCCAA
CATGGTGAAACCCCATCTCTACTAAAAATACAAAATTAGCCGGGCGTAGTGGCGGGCGCCTGTAA
TCCCA
GCTACTTGGGAGGCTTAGGCAGGAGAATCGCTTGAACCTGGGAGGCGGAGGTTGTAGTGAGCCGA
GATTG
CACCATTGCACTCCAGCCTGGGTGACAAAAGCAAAAACTCCGTCTCAAAAAAAAAAGAATTAGCC
AGGGG
TAGTGGTGAACGCCTGTAGTCCCAGCTACTCAGGAGGCAGAGGCAGGAGAATCACTTGAACCCCG
GAGGC
AGAGGTTGCAGTGAGCCGAGATTGTCCCATTGCACTCCAGCCTAGGCGAGAAGAGCAAAATTCCA
TGTCA
AAAAAAAAAAAAAAAAAGGAAAGAAAAAAAATAACGATTAGAAAGGAAGAAATCAAACACATT
CACAGCC
AGTATGATTCTATACATACCATGGTCCTAATGGGGCCAGGCGTGGTGGCTCATGCTGTAATCCTAG
CACT
TTTAGGAGGCTGAGGCAGGTGGCTTCCCTGGGACCAGCTGGCCAACATGGTGAAACCCCAACTCT
AATAA
AAATACAAAAAATCAGCCAGGCGTGGTGAGGGCACCTCTAATCCCAGCTACTCAGGAGGCTGAGG
CAGGA
GAATTGCTTGGACCTGGGAGGCAGAGGTTGCAGTGAGCCGAGATCGCGCTATTGCACTCCAGCCT
GGGCA
ACAAGAGTGAAACTCCGGCAGGGTGTGGTCTTACGCCTGTAATCCCAGCACTTCGGGAGGCTGAG
CCAGG

CCGATCACCTGAGGTCAGGAGTTTGAGACCAACCTAACATGGTGAAACCCCGTCTCTACTAAAAAT
ACAA
GAATTAGCTGGGTGTAGTGGTGGGCGCCTGTAATCCCAGCTACTTGGGAGGCTGAGACAGAAGAA
TTGCT
TGAACCCAGGAGGTGGAGGTTGCAGTGAGCTGAGATCATGCCATTGCACACCACGCCGGGCAACA
GAGCG
AGATTCCGTCTCAAAAAAAAAAAAAAAGATGAAACTCTATCTCAAAAAAAAAAAAAAAGTCCTAATG
GAAAA
TCCATAAAAAGCTACCAAAACTAATAAATAAATATAGCAGGGTTGCAGGTTACAGGGCAATATAG
TTATC
CCTCTATCTGTAGGGGCTTGGTTCTGGGACTCCTCACACACCAAACCCACAGATGTCTAAGTCCCA
TATA
TAAGACGGAATAGTATTTAACCTACACATATCCTCCCATATAGTTTAAATTATCTAGATTACTTACA
TTA
CCCCCATACAATGAAAATGCTAATGTACATGCAAGTATGTATGTAAGTACTTGTACTATATTGTTT
AGGG
AATCACTGGACAGATAGGCCTTCAAGACTGATACCAGCAGCCACTGTTAAGATTCTGGTCAGGCCT
GCCC
CTGTTTGGGGTCTCAGTTGATCTCATTGCCTTCCCACCCAGCCAAGGGCACCTGCATTTCTCTTGGC
TCC
CTGGCCATTTGGAAGGCCTAGTTCAGCCTGGCACATTTGTATCCTGGCCCACTGATGCTGGTACCC
CTGG
GAAGGTCCTGCTCTGAAAAACACGGAGATTTTAGTTGCTACTGAAGATTTGAGAGATAAAGACAG
GGAGA
CCTGTCTGTAGACCTGTGTCCCTCCAAGTGGGATTGAGACTTTGGGCCCCCCATTTCAGGACAGCA
CCTC
CTGGCCTGTTGACTGAATAGATCCCTGAAGGAGGTGTAGTTGCATTTTAGGAGTGGGGGTGGGAG
CAGTA
CCACTGATCCGCACTAACAATCACACAGTTCTCTCTAGAATAATAATATAGAACAAGTGAAATAG
AACAA
TTGCAGAAAGAGCTAACCTTTGTTGAGCTCTTACTGTGTGCCCAGCACTTTCCTCAACTCTACATTT
CCC
ATAATACATAGAGTACTAGGTAGGCGGGGCTTGGGGGCTCACGCCTGTAATCCCAGCACTTTAGG
AGGCC
AAGGGGGGTGGATCACCTGAGGTCGGGAGTTCAAGACCAGCCTGACTAACATGGTGAAACCCCGT
CTCTA
CTAGAAGTACAAAATTAGCCAGGTGTGGTGGCACATGCTTGTAGTCCTAGCTACTCAGCAGGCTGA
GGCA
GGAGAATCATTTGAATCCGGGAGGAGGTTGCAGTAAGCGGAGATAGTGCCACTGTACTCCAGCCT
GGGCA
ATAAGAGCTGAGACTCCGTCTCAAAATAAAATAAAATAAAATAAAATAAAATAAAATAAAATAA
AAAAAG
AAAAGAGCCTGCCATTAAAGGAGCTGTTTGGTAGGGGATGTTTTGTCAGTGCAAACAACAGAAAA
GTGGG

CTGGGCACAGTGGTTCATGCCTGTAATCCCAGCACTTTGGGAGGCCAAGGCGGGCGGATCACCTG
AAGTT

GGGAGTTCAAGACCAGCCTGACCAATATGGAGAAACCCCGTCTCTACTAAAAATACAAAATTAGC
CGGGC

GCAGTGGCCGATGCCTGTAATCCCAGCTACTCGGGAGGCTGAGGCAGGAGAATCGCTTGAACCTG
GGAGG

CAGAGGTTGCGGTGAGCCGAGATCGCACCATTGCACTCCAGCCTGGACGAGAGCAAAACTCTGTC
TCAAA

AAAAAAAAAAAACAGAAAAGTGTAACAAACACTTACAGTAGGCATGTTTCTTAGCAAATCTGATG
ACAAA

TTTGGCATAAAGAAAGAGAGCATCCCTGAAAAAAAAAAAAAGAAAAAGAAAGAGAGCATCCTGC
CTGGGC

AACATAGTGAAACCCTGCCTCTACAAAAAAACTCAAAAATTGGCCGGGTGCAGTGGCTCACACCT
GTAAT

CCCAGCACTTTGGGAGTCGGAGGCGGGAGGATCACCTGAGGTCAGGAGTTCGAAACCAGCCTGGC
CAACA

TGGCAAAACCCCATCTCTACTAAAAATACAAAAAATTAATCAGGCGCATTGGTGGGCGCCTGTAA
TCCCA

GCTACTCAGGAAGTTGAGGCAAGAGGATCGCTTGATACTGGGAGGTGGAGGTTACAGTGAGTCGA
GATCA

CACCACTGCACTCTAGCCTGGGTGACAGGGCGAGACTCCGTCTCCAAAAAAAAAAAGAAAAAGAA
AAAGA

CTAAAAAATTAGCCAGGCAGGCCTCTGTGGTCCCAGCTACTTGGGAGGCTGAGGCAGGAGAATCA
CTGAG

CCCAGGAGTGGCAGGCTGTAGTGAGCCATGATTGCACCACTGTACCCTAGCTTGGGCTTCAAAGCA
AGAC

CCTGCCTCAAAAGAAAAAAGAAAGAAAGAAAGAACATGGCGGGCCAGGCACAGTGGCTCACACC
TGTAAT

CCCAGCGCTTTGAGAGGCCGAGGCAGGTGGATCACAAGGTCAGGAGTTCCACACCAGCCTGGCCA
ACATG

GTGAAACCCTGTCTCTACTAAAAATACAAAAAATCAGCAGGCAGGGTGGTAGGGGCCTGTAATCC
CAGCT

ACTCGGGAGGCTGAGGCAGGAGAATTGCTTGAAACCAGAAGGCAGAGGTTGCAGTGAGCCTAGA
CTGCAC

CACTGCACTCCAGCCTGGGCGAAAAGAGCCAAACTCCATCTCAAAAAACAAACAAAAAAACAAA
ACAAAA

AAAAACATGGCAGCCTTTGAAAGCTTGTCTGGGAGAAGGTGCGATGATGGTTGCATAACTTCGTG
CAAGA

TGCTGGTCCACACAGGGGCTGCCCCTTGCTCTTTCTCGCTCTCTTAACCTCTCATATAACAGGCTTG
TGT

GTTATGCACATTTATTGAGCCCAAGCAGGTGCAAGGCATTGTGATCTAATACTTTGGTCAGCAAGA
CAAC

AAGATAGATCACTGCCCTGCCCTTAGGAAGTGTATATGCTATTAGAGGAAACAGATAAAATAAAC
AAGGA

AAAGTATCAGACAATGTAAGTGCTATGAGAATGCAAATGAGGTGATGTGAATTAAAATAGGATGA
CTTAA
GTCTGCACGGAAGGCCCCTACCCCCATGTTCCTGGCTAGCCAAGGAACCACCAGTTGATTAGCAGA
GAAG
GGCAGCCCGTCTAGCTAGAGCTTTTGGGGAAGAGGGAGTGGTTGTTAAGAGATGAGATTAAAGAA
GCCGA
GACGGGCCCTTCGTGAGGGGGGGTTGTAATGCAGGGCTGAGGAGTGTCCGAAGAGAATGGGCAG
GTGAGC
GGTGAGACAGTTGTTCTTCCAGAAGCTTTGCAGTGAAAGGAATCAAAGAAATGGAGCCGTGTATC
AGGTG
GGGAAGGGTGGGGGCCAAGGGGGTGTCCTTCCCCATACAGAGATTGCAGGCTGAGAATGACTATA
TCCTT
GTTAACAGGAGGTGGGAGCAGGGCACGGTAGCTCACACCTGTAATCTTGGCACTTTAGGAGGCGG
AGGCG
GGCCGATCACCTGAAGTAAGGAGTTCGAGACCAGCCTGGCCAACATGCAAAGCCCTGTCTCTACT
AAAAA
TACAAAAATTAGCTGGGTGTGGTGGTACTCGCCTGTAATCCCAGCTACTCGGGAGACTGAGGCAG
GAGAA
TGGCTTGAACCCGGAAGGTAGAGGTTGCAGTGAGCTGAGATCATGCCACTGTGCTCCAGCCTAGG
TGACA
GAGAGAGACTCCATCTCAAAAAAAAAAAAAAAATACAGGAAGGGAGTTGGGAATAGGGTGCACAT
TTAGGA
AGTCTTGGGGATTTAGTGGTGGGAAGGTTGGAAGTCCCTCTCTGATTGTCTTTTCCTCAAAGAAGT
GCAT
GGCTGGTGTGGGGTGGGGCAGGAGTGCTTGGGTTGTGGTGAAACATTGGAAGAGAGAATGTGAAG
CAGCC
ATTCTTTTCCTGCTCCACAGGAAGCCGAGCTGTCTCAGACACTGGCATGGTGTTGGGGGAGGGGGT
TCCT
TCTCTGCAGGCCCAGGTGACCCAGGGTTGGAAGTGTCTCATGCTGGATCCCCACTTTTCCTCTTGCA
GCA
GCCAGACTGCCTTCCGGGTCACTGCCATGGAGGAGCCGCAGTCAGATCCTAGCGTCGAGCCCCCTC
TGAG
TCAGGAAACATTTTCAGACCTATGGAAACTGTGAGTGGATCCATTGGAAGGGCAGGCCACCACCC
CGACC
CCAACCCCAGCCCCCTAGCAGAGACCTGTGGGAAGCGAAAATTCATGGGACTGACTTTCTGCTCTT
GTCT
TTCAGACTTCCTGAAAACAACGTTCTGGTAAGGACAAGGGTTGGGCTGGGGACCTGGAGGGCTGG
GGGGC
TGGGGGGCTGAGGACCTGGTCCTCTGACTGCTCTTTTCACCCATCTACAGTCCCCCTTGCCGTCCCA
AGC
AATGGATGATTTGATGCTGTCCCCGGACGATATTGAACAATGGTTCACTGAAGACCCAGGTCCAGA
TGAA
GCTCCCAGAATGCCAGAGGCTGCTCCCCGCGTGGCCCCTGCACCAGCAGCTCCTACACCGGCGGCC
CCTG

CACCAGCCCCCTCCTGGCCCCTGTCATCTTCTGTCCCTTCCCAGAAAACCTACCAGGGCAGCTACG
GTTT
CCGTCTGGGCTTCTTGCATTCTGGGACAGCCAAGTCTGTGACTTGCACGGTCAGTTGCCCTGAGGG
GCTG
GCTTCCATGAGACTTCAATGCCTGGCCGTATCCCCCTGCATTTCTTTTGTTTGGAACTTTGGGATTC
CTC
TTCACCCTTAGGCTTCCTGTCAGTGTTTTTTTATAGTTTACCCACTTAATGTGTGATCTCTGACTCCT
GT
CCCAAAGTTGAATATTCCCCCCTTGAATTTGGGCTTTTATCCATCCCATCACACCCTCAGCATCTCT
CCT
GGGGATGCAGAACTTTTCTTTTTCTTCATCCACGTGTATTCCTTGGCTTTTGAAAATAAGCTCCTGA
CCA
GGCTTGGTGGCTCACACCTGCAATCCCAGCACTCTCAAAGAGGCCAAGGCAGGCAGATCACCTGA
GCCCC
AGGAGTTCAAGACCAGCCTGGGTAACATGATGAAACCTCGTCTCTACAAAAAAATACAAAAAATT
AGCCA
GGCATGGTGGTGCACACCTATAGTCCCAGCCACTCAGGAGGCTGAGGTGGGAAGATCACTTGAGG
CCAGG
AGATGGAGGCTGCAGTGAGCTGTGATCACACCACTGTGCTCCAGCCTGAGTGACAGAGCAAGACC
CTATC
TCAAAAAAAAAAAAAAAAGAAAAGCTCCTGAGGTGTAGACGCCAACTCTCTCTAGCTCGCTAGTGG
GTTGC
AGGAGGTGCTTACACATGTTTGTTTCTTTGCTGCCGTGTTCCAGTTGCTTTATCTGTTCACTTGTGCC
CT
GACTTTCAACTCTGTCTCCTTCCTCTTCCTACAGTACTCCCCTGCCCTCAACAAGATGTTTTGCCAA
CTG
GCCAAGACCTGCCCTGTGCAGCTGTGGGTTGATTCCACACCCCCGCCCGGCACCCGCGTCCGCGCC
ATGG
CCATCTACAAGCAGTCACAGCACATGACGGAGGTTGTGAGGCGCTGCCCCCACCATGAGCGCTGC
TCAGA
TAGCGATGGTGAGCAGCTGGGGCTGGAGAGACGACAGGGCTGGTTGCCCAGGGTCCCCAGGCCTC
TGATT
CCTCACTGATTGCTCTTAGGTCTGGCCCCTCCTCAGCATCTTATCCGAGTGGAAGGAAATTTGCGTG
TGG
AGTATTTGGATGACAGAAACACTTTTCGACATAGTGTGGTGGTGCCCTATGAGCCGCCTGAGGTCT
GGTT
TGCAACTGGGGTCTCTGGGAGGAGGGGTTAAGGGTGGTTGTCAGTGGCCCTCCGGGTGAGCAGTA
GGGGG
GCTTTCTCCTGCTGCTTATTTGACCTCCCTATAACCCCATGAGATGTGCAAAGTAAATGGGTTTAAC
TAT
TGCACAGTTGAAAAAACTGAAGCTTACGAGGCTAAGGGCCTCCCCTGCTTGGCTGGGCGCAGTGG
CTCAT
GCCTGTAATCCCAGCACTTTGGGAGGCCAAGGCAGGCGGATCACGAGGTTGGGAGATCGAGACCA
TCCTG

GCTAACGGTGAAACCCCGTCTCTACTGAAAAATACAAAAAAAAATTAGCCGGGCGTGGTGCTGGG
CACCT

GTAGTCCCAGCTACTCGGGAGGCTGAGGAAGGAGAATGGCGTGAACCTGGGCGGTGGAGCTTGCA
GTGAG

CTGAGATCACGCCACTGCACTCCAGCCTGGGCGACAGAGCGAGATTCCATCTCAAAAAAAAAAAA
AAAAG

GCCTCCCCTGCTTGCCACAGGTCTCCCCAAGGCGCACTGGCCTCATCTTGGGCCTGTGTTATCTCCT
AGG

TTGGCTCTGACTGTACCACCATCCACTACAACTACATGTGTAACAGTTCCTGCATGGGCGGCATGA
ACCG

GAGGCCCATCCTCACCATCATCACACTGGAAGACTCCAGGTCAGGAGCCACTTGCCACCCTGCACA
CTGG

CCTGCTGTGCCCCAGCCTCTGCTTGCCGCTGACCCCTGGGCCCACCTCTTACCGATTTCTTCCATAC
TAC

TACCCATCCACCTCTCATCACATTTCCGGCGGGAATCTCCTTACTGCTCCCACTCAGTTTCCTTTTCT
CT

GGCTTTGGGACCTCTTAACCTGTGGCTTCTCCTCCCACCTCCTGGAGCTGGAGCTTAGGCTCCAGA
AAGG

ACAAGGGTGGTTGGGAGTAGATGGAGCCTGGTTTTTTAAATGGGACAGGTAGGACCTGATTTCCTT
ACTG

CCTCTTGCTTCTCTTTTCCTATCCTGAGTAGTGGTAATCTACTGGGACGGAACAGCTTTGAGGTGCG
TGT

TTGTGCCTGTCCTGGGAGAGACCGGCGCACAGAGGAAGAGAATCTCCGCAAGAAAGGGGAGCCTC
ACCAC

GAGCTGCCCCCAGGGAGCACTAAGCGAGGTAAGCAAGCAGGACAAGAAGCGGTGGAGGAGACCA
AGGGTG

CAGTTATGCCTCAGATTCACTTTTATCACCTTTCCTTGCCTCTTTCCTAGCACTGCCCAACAACACC
AGC

TCCTCTCCCCAGCCAAAGAAGAAACCACTGGATGGAGAATATTTCACCCTTCAGGTACTAAGTCTT
GGGA

CCTCTTATCAAGTGGAAAGTTTCCAGTCTAACACTCAAAATGCCGTTTTCTTCTTGACTGTTTTACC
TGC

AATTGGGGCATTTGCCATCAGGGGGCAGTGATGCCTCAAAGACAATGGCTCCTGGTTGTAGCTAAC
TAAC

TTCAGAACACCAACTTATACCATAATATATATTTTAAAGGACCAGACCAGCTTTCAAAAAGAAAAT
AGTT

AAAGAGAGCATGAAAATGGTTCTATGACTTTGCCTGATACAGATGCTACTTGACTTACGATGGAGT
TACT

TCTGATAACTCGTCGTAAGTTGAAATATTGAAATATTGTAAGTTGAAAATGGATTTAATACACCTA
ATCT

AAGGAACATCATAGCTTAGCCTAGCCTGCTTTTTTTTTTTTTTTTTTTTGGAGACAGAGTCTCACTC
TG

CTACCCAGGCTGGAGTGCAGTGGCGGGATCTCGGCTCACTGCAACCTCCGCCTTCTGGGTTCAAGC
GATT

CTCCTGCCTCAGCCCACTGAGTAGCTGGGATTACAGGCACCTGCCCCGACGCCCAGCTAATTTTTT
GTTA
TTTATTTCCTTTTTTTTTAGTAGAGATAGAATTTCACCATGTTGGCCAGGCTAGTCTCGAACTCCTG
ACC
TTGTGATCTGCCTGCCTTGGCCTCCCAAAGTGCTGGGATTACAGGCGTGAGCCACCGCACCTGGCC
TGCC
TAGCCTACTTTTATTTTATTTTTAATGGAGACAGCATCTTGCTCTGTTGCCCAGGCTGGATTACAGT
GAT
GTGATCATAGCTCATTATACCCTCCTGGGCTCAAGCAATCCCCCTAACTCTGCCTCCCCAGTAGCTA
GGA
CCACAGGCATACACCACCATACCCAGCTAATTTTTAAAATTTTTTGTAGATAGATAGAGTCTCACT
ATGT
TGCCCAGGCTGGTCTCTAGCCTACTTTTTTGAGACAAGGTCTTGCTCTGTCACCCAGGCTGGATAG
AGTG
CAGTAGTGCAGTCACAGCTCACTGCAGCCTCCACCTCCCAGGCTCCATCCATCCTCCCAGCTCAGC
CTCC
CAAGTTGCTTCAACTACAGGCCTGCACCACCATGCCTGGCTAATTTTTATTTATTTATTTTTATTTTA
TT
TTATTTTATTTTTTGAGACTCAGTCTCACTCTGTCGCCTTAGGCTGGAGTGCAGTGGCATGATCTCG
GCT
CACTGCTAACCTCTGCCTCCTGGGTTTCAAGTGATTCTCCTGCCTCAGCCTCCCGAATAGCTAGGAC
TAC
AAGCGCCTGCTACCACGCCCGGCTAATTTGTGTATTTTTAGTAGAGACAGGGTTTCACCATGTTGG
CCAG
GCTGGTCTCGAACTTCTGACCATGTGATCGCCGCCTCGGCCTCCCAAAGTGCTGGGATTACAGGTG
TGAG
CCACCACGCCCGGCTAATTTTTATTTATTTATTTAAAGACAGAGTCTCACTCTGTCACTCAGGCTAG
AGT
GCAGTGGCACCATCTCAGCTCACTGCAGCCTTGACCTCCCTGGGCTCCGGTGATTTCACCCTCCCA
AGTA
GCTAGGACTACAGGCACATGCCACGACACCCAGCTAATTTTTTATTTTCTGTGAAGTCAAGGTCTT
GCTA
CGTTGCCCATGCTGGTATCAAACCCCTGGGCTCAATCAATCCTTCCACCTCAGCCTCCCCAAGTATT
GGG
GTTACAGGCATGAGCTACCACACTCAGCCCTAGCCTACTTGAAACGTGTTCAGAGCATTTAAGTTA
CCCT
ACAGTTGGGCAAAGTCATCTAACACAAAGCCCTTTTTATAGTAATAAAATGTTGTATATCTCATGT
GATT
TATTAGATATTGTTACTAAAAGTGAGAAACAGCATGGTTGCATGAAAGGAGGCACAGTCGAAGCC
AGGCA
CAGCCTGGGCGCAGAGCGAGACTCAAAAAAAGAAAAGGCCAGGCGCACTCTCACGCCTGTAATCC
CAGCA
TTTCGGGAGGCTGAGGCGGGTGGATCACCTGAGGTCAGGAGTTCAAGACCAGCCTAGCCAACATG
GTGAA

ACCCCGTCTCTACTAAAATACAAAAATTAACCGGGCGTGATGGCAGGTGCCTGTAATCCCAGCTAC
TTGG
GAGGCTGAGGCAGGAGAATCGCTTGAACCAGGAGGCGGAGGTTGCAGGGAGCCAAGACGGCGCC
ACTGCA
CTCCAGCCTGGGCGATAGAGTGAGACTCCGTCTCAGAAAAAAAAGAAAAGAAACGAGGCACAGT
CGCATG
CACATGTAGTCCCAGTTACTTGAGAGGCTAAGGCAGGAGGATCTCTTGAGCCCAAGAGTTTGAGTC
CAGC
CTGAACAACATAGCAAGACATCATCTCTAAAATTTAAAAAAGGGCCGGGCACAGTGGCTCACACC
TGTAA
TCCCAGCACTTTGGGAGGTGGAGGTGGGTAGATCACCTGACGTCAGGAGTTGGAAACCAGCCTGG
CTAAC
ATGGTGAAGCCCCATCTCTACTAAAAACACAAAAATTAGCCAGTGTGAGACACGTTGAGTCCACG
TACTC
GGAGGCTGAGGCACAAGAATCACTTGAACCCCAGAGGCGGAGATTCGAATCAGCCAAGATTGCAC
CATTG
CACTCCCGCCTGGGCGACGAGAGTGAGACCCCATCTCAAAATAAATAAATAAATATTTTTAAAAG
TCAGC
TGTATAGGTACTTGAAGTGCAGTTTCTACTAAATCGATGTTGCTTTTGATCCGTCATAAAGTCAAAC
AAT
TGTAACTTGAACCATCTTTTAACTCAGGTACTGTGTATATACTTACTTCTCCCCCTCCTCTGTTGCTG
CA
GATCCGTGGGCGTGAGCGCTTCGAGATGTTCCGAGAGCTGAATGAGGCCTTGGAACTCAAGGATG
CCCAG
GCTGGGAAGGAGCCAGGGGGGAGCAGGGCTCACTCCAGGTGAGTGACCTCAGCCCCTTCCTGGCC
CTACT
CCCCTGCCTTCCTAGGTTGGAAAGCCATAGGATTCCATTCTCATCCTGCCTTCATGGTCAAAGGCA
GCTG
ACCCCATCTCATTGGGTCCCAGCCCTGCACAGACATTTTTTTAGTCTTCCTCCGGTTGAATCCTATA
ACC
ACATTCTTGCCTCCACGTAGTATCCACAGAACATCCAAACCCAGGGACGAGTGTGGATACTTCTTT
GCCA
TTCTCCGCCAACTCCCCAGCCCAGAGCTGGAGGGTCTCAAGGGGCCTAATAATTGTGTAATACTGA
ATAC
AGCCAGAGTTTCAGGTCATATACTCAGCCCTGCCATGCACCGGCAGGTCCTAGGTGACCCCCGTCA
AACT
CAGTTTCCTTATATATAAAATGGGGTAAGGGGGCCGGGCGCAGTGGCTCACGAATCCCACACTCTG
GGAG
GCCAAGGCGAGTGGATCACCTGAGGTCGGGAGTTTGAGCCCAGCCTGACCAACATGGAGAAACCC
CATCT
CTACTAAAAATACAAAAGTAGCCGGGCGTGGTGATGCATGCCTGTAATCCCAGCTACCTACTCGG
GAGGC
TGAGGCAGGAGAATCGCTTGAACCCGGGAGGCAGAGGTTGCGGTGAGCTGAGATCTCACCATTAC
ACTCC

AGCCTGGGCAACAAGAGTGAAACTCCGTCTCAAAAAAGTATAATAAAGTAAAATGGGGTAAGGG
AAGATT
ACGAGACTAATACACACTAATACTCTGAGGTGCTCAGTAAACATATTTGCATGGGGTGTGGCCACC
ATCT
TGATTTGAATTCCCGTTGTCCCAGCCTTAGGCCCTTCAAAGCATTGGTCAGGGAAAAGGGGCACAG
ACCC
TCTCACTCATGTGATGTCATCTCTCCTCCCTGCTTCTGTCTCCTACAGCCACCTGAAGTCCAAAAAG
GGT
CAGTCTACCTCCCGCCATAAAAAACTCATGTTCAAGACAGAAGGGCCTGACTCAGACTGACATTCT
CCAC
TTCTTGTTCCCCACTGACAGCCTCCCACCCCCATCTCTCCCTCCCCTGCCATTTTGGGTTTTGGGTCT
TT
GAACCCTTGCTTGCAATAGGTGTGCGTCAGAAGCACCCAGGACTTCCATTTGCTTTGTCCCGGGGC
TCCA
CTGAACAAGTTGGCCTGCACTGGTGTTTTGTTGTGGGGAGGAGGATGGGGAGTAGGACATACCAG
CTTAG
ATTTTAAGGTTTTTACTGTGAGGGATGTTTGGGAGATGTAAGAAATGTTCTTGCAGTTAAGGGTTA
GTTT
ACAATCAGCCACATTCTAGGTAGGTAGGGGCCCACTTCACCGTACTAACCAGGGAAGCTGTCCCTC
ATGT
TGAATTTTCTCTAACTTCAAGGCCCATATCTGTGAAATGCTGGCATTTGCACCTACCTCACAGAGTG
CAT
TGTGAGGGTTAATGAAATAATGTACATCTGGCCTTGAAACCACCTTTTATTACATGGGGTCTAAAA
CTTG
ACCCCCTTGAGGGTGCCTGTTCCCTCTCCCTCTCCCTGTTGGCTGGTGGGTTGGTAGTTTCTACAGT
TGG
GCAGCTGGTTAGGTAGAGGGAGTTGTCAAGTCTTGCTGGCCCAGCCAAACCCTGTCTGACAACCTC
TTGG
TCGACCTTAGTACCTAAAAGGAAATCTCACCCCATCCCACACCCTGGAGGATTTCATCTCTTGTAT
ATGA
TGATCTGGATCCACCAAGACTTGTTTTATGCTCAGGGTCAATTTCTTTTTTCTTTTTTTTTTTTTTTT
T
CTTTTTCTTTGAGACTGGGTCTCGCTTTGTTGCCCAGGCTGGAGTGGAGTGGCGTGATCTTGGCTTA
CTG
CAGCCTTTGCCTCCCCGGCTCGAGCAGTCCTGCCTCAGCCTCCGGAGTAGCTGGGACCACAGGTTC
ATGC
CACCATGGCCAGCCAACTTTTGCATGTTTTGTAGAGATGGGGTCTCACAGTGTTGCCCAGGCTGGT
CTCA
AACTCCTGGGCTCAGGCGATCCACCTGTCTCAGCCTCCCAGAGTGCTGGGATTACAATTGTGAGCC
ACCA
CGTGGAGCTGGAAGGGTCAACATCTTTTACATTCTGCAAGCACATCTGCATTTTCACCCCACCCTTC
CCC
TCCTTCTCCCTTTTTATATCCCATTTTTATATCGATCTCTTATTTTACAATAAAACTTTGCTGCCACC
TG

TGTGTCTGAGGGGTGAACGCCAGTGCAGGCTACTGGGGTCAGCAGGTGCAGGGGTGAGTGAGGAG
GTGCT
GGGAAGCAGCCACCTGAGTCTGCAATGAGTGTGGACTGGGGGCCCAGTGCCCGGGTTCCGGGAG
GGGAA
CAAAGGCTGGAGACTGGGTCAGTCTGCGGGCTGCATGACAACAAGGGAGGGGGTGGCTCCATTCA
TAACT
CAGGAACCAACCGTCCCTCCTCCCCTCCGCCCACGGCTGGCACAAGGTTCTCTGCCTCCCCTGCTTC
TAG
GATTGGGCTGCTTCCCCCTCGGCAGCCTCTCACCAAGGATTACGGATTTAAATGTCGTGATTTAC
GAAG
GCTGAGCCTCCAGGGTGGCCATCTTCGTCCATCAGAAGTGGCAGGATACCTGGGTTCCAAGGGAA
CAGGG
TGG

P53 (Forward)<br><br>*P53* (Reverse)	TGTGTCTGAGGGGTGAACG<br><br>GGACGGTTGGTTCCTGAGTTA
Reverse compliment for reverse primer:	TAACTCAGGAACCAACCGTCC

Homo sapiens heat shock protein 70 (hsp70) mRNA, complete cds

GenBank: L12723.2

GenBank Graphics

>L12723.2 Homo sapiens heat shock protein 70 (hsp70) mRNA, complete cds
CTAAAGCCGCCGGGGGTCCGTGTCCTGTCTCGGTTGGCCGGACCCGGGCCCGAGCCCGAGCAGTA
GCCGG
CGCCATGTCGGTGGTGGGCATAGACCTGGGCTTCCAGAGCTGCTACGTCGCTGTGGCCCGCGCCGG
CGGC
ATCGAGACTATCGCTAATGAGTATAGCGACCGCTGCACGCCGGCTTGCATTTCTTTTGGTCCTAAG
AATC
GTTCAATTGGAGCAGCAGCTAAAAGCCAGGTAATTTCTAATGCAAAGAACACAGTCCAAGGATTT
AAAAG
ATTCCATGGCCGAGCATTCTCTGATCCATTTGTGGAGGCAGAAAAATCTAACCTTGCATATGATAT
TGTG
CAGTGGCCTACAGGATTAACAGGTATAAAGGTGACATATATGGAGGAAGAGCGAAATTTTACCAC
TGAGC
AAGTGACTGCCATGCTTTTGTCCAAACTGAAGGAGACAGCCGAAAGTGTTCTTAAGAAGCCTGTA
GTTGA
CTGTGTTGTTTCGGTTCCTTGTTTCTATACTGATGCAGAAAGACGATCAGTGATGGATGCAACACA
GATT
GCTGGTCTTAATTGCTTGCGATTAATGAATGAAACCACTGCAGTTGCTCTTGCATATGGAATCTAT
AAGC

AGGATCTTCCTCGCTTAGAAGAGAAACCAAGAAATGTAGTTTTTGTAGACATGGGCCACTCTGCTT
ATCA

AGTTTCTGTATGTGCATTTAATAGAGGAAAACTGAAAGTTCTGGCCACTGCATTTGACACGACATT
GGGA

GGTAGAAAATTTGATGAAGTGTTAGTAAATCACTTCTGTGAAGAATTTGGGAAGAAATACAAGCT
AGACA

TTAAGTCCAAAATCCGTGCATTATTACGACTCTCTCAGGAGTGTGAGAAACTCAAGAAATTGATGA
GTGC

AAATGCTTCAGATCTCCCTTTGAGCATTGAATGTTTTATGAATGATGTTGATGTATCTGGAACTATG
AAT

AGAGGCAAATTTCTGGAGATGTGCAATGATCTCTTAGCTAGAGTGGAGCCACCACTTCGTAGTGTT
TTGG

AACAAACCAAGTTAAAGAAAGAAGATATTTATGCAGTGGAGATAGTTGGTGGTGCTACACGAATC
CCTGC

GGTAAAAGAGAAGATCAGCAAATTTTTCGGTAAAGAACTTAGTACAACATTAAATGCTGATGAAG
CTGTC

ACTCGAGGCTGTGCATTGCAGTGTGCCATCTTATCGCCTGCTTTCAAAGTCAGAGAATTTTCTATCA
CTG

ATGTAGTACCATATCCAATATCTCTGAGATGGAATTCTCCAGCTGAAGAAGGGTCAAGTGACTGTG
AAGT

CTTTTCCAAAAATCATGCTGCTCCTTTCTCTAAAGTTCTTACATTTTATAGAAAGGAACCTTTCACT
CTT

GAGGCCTACTACAGCTCTCCTCAGGATTTGCCCTATCCAGATCCTGCTATAGCTCAGTTTTCAGTTC
AGA

AAGTCACTCCTCAGTCTGATGGCTCCAGTTCAAAAGTGAAAGTCAAAGTTCGAGTAAATGTCCATG
GCAT

TTTCAGTGTGTCCAGTGCATCTTTAGTGGAGGTTCACAAGTCTGAGGAAAATGAGGAGCCAATGGA
AACA

GATCAGAATGCAAAGGAGGAAGAGAAGATGCAAGTGGACCAGGAGGAACCACATGTTGAAGAGC
AACAGC

AGCAGACACCAGCAGAAAATAAGGCAGAGTCTGAAGAAATGGAGACCTCTCAAGCTGGATCCAA
GGATAA

AAAGATGGACCAACCACCCCAATGCCAAGAAGGCAAAAGTGAAGACCAGTACTGTGGACCTGCC
AATCGA

GAATCAGCTATATGGCAGATAGACAGAGAGATGCTCAACTTGTACATTGAAAATGAGGGTAAGAT
GATCA

TGCAGGATAAACTGGAGAAGGAGCGGAATGATGCTAAGAACGCAGTGGAGGAATATGTGTATGA
AATGAG

AGACAAGCTTAGTGGTGAATATGAGAAGTTTGTGAGTGAAGATGATCGTAACAGTTTTACTTTGAA
ACTG

GAAGATACTGAAAATTGGTTGTATGAGGATGGAGAAGACCAGCCAAAGCAAGTTTATGTTGATAA
GTTGG

CTGAATTAAAAAATCTAGGTCAACCTATTAAGATACGTTTCCAGGAATCTGAAGAACGACCAAATT
ATTT

GAAGAACTAGAGAACTTAGGGAAACAGATCCAACAGTATATGAAAATAATCAGCTCCTTTCAAAA ACAAG

GAGGACCAGGTATGATCATTTGGATGCTGCTGACATGACAAAGGTAGAAAAAAGCACAAATGAAG CAATG

GAGTGGATGGAAGTCACACCAA

HSP70 (Forward)<br>*HSP70* (Reverse)	TCCAGGAATCTGAAGAACGACC<br>GGTGTGACTTCCATCCACTC
Reverse compliment for reverse primer:	GAGTGGATGGAAGTCACACC
